# AI in Histopathology Explorer for comprehensive analysis of the evolving AI landscape in histopathology

**DOI:** 10.1038/s41746-025-01524-2

**Published:** 2025-03-12

**Authors:** Yingrui Ma, Shivprasad Jamdade, Lakshmi Konduri, Heba Sailem

**Affiliations:** 1 School of Cancer and Pharmaceutical Sciences, Stamford St., Franklin Wilkins Building, King’s College London, London, UK; 2 King’s Institute for Artificial Intelligence, King’s College London, London, UK

**Keywords:** Biomarkers, Mathematics and computing

## Abstract

Digital pathology and artificial intelligence (AI) hold immense transformative potential to revolutionize cancer diagnostics, treatment outcomes, and biomarker discovery. Gaining a deeper understanding of deep learning algorithm methods applied to histopathological data and evaluating their performance on different tasks is crucial for developing the next generation of AI technologies. To this end, we developed AI in Histopathology Explorer (HistoPathExplorer); an interactive dashboard with intelligent tools available at www.histopathexpo.ai. This real-time online resource enables users, including researchers, decision-makers, and various stakeholders, to assess the current landscape of AI applications for specific clinical tasks, analyze their performance, and explore the factors influencing their translation into practice. Moreover, a quality index was defined for evaluating the comprehensiveness of methodological details in published AI methods. HistoPathExplorer highlights opportunities and challenges for AI in histopathology, and offers a valuable resource for creating more effective methods and shaping strategies and guidelines for translating digital pathology applications into clinical practice.

## Introduction

Histopathology plays an important role in cancer patient diagnosis where pathologists infer several clinical features based on changes in tissue architecture and cellular traits. Moreover, many studies have shown that morphological patterns can be predictive of molecular traits, treatment response, and even survival^1,2^. These results motivated the development of a suite of artificial intelligence (AI) powered systems to improve the precision and efficiency of cancer diagnosis and detection from histopathology images, facilitating timely interventions. A major advantage of AI algorithms is that they can rapidly detect patterns in tissue architecture and cellular traits by analyzing thousands of gigapixel-sized images with millions of visual features. AI can also automate time-consuming tasks such as detecting the number of mitotic cells or cells positive for a certain marker, such as PD-L1, in an image, reducing the time and effort required for analysis and reporting^3–5^. Most importantly, it holds promise for personalised medicine by identifying biomarkers and predicting treatment response, ultimately leading to more tailored treatment plans.

In clinical practice, the most widely used form of histopathological data is whole slide images (WSI). The dimensions of these images can reach millions of pixels. Tumors might occupy only a small region of the imaged tissue, posing challenges for annotation and pattern recognition. To address this and to obtain slide-based representation, machine and deep learning methods divide WSIs into smaller patches that can be fed directly into deep learning models and then aggregated^6,7^. Recent weakly supervised learning approaches, such as Vision Transformers (ViTs) or Dual-Stream Multiple Instance Learning (DSMIL) enable performing slide-level analysis by converting patches into feature vectors using pre-trained or self-supervised models such as RetCCL^8,9^. These will be fed as a sequence of patch feature vectors to a deep learning model which will learn to aggregate the patches specific to the task. These approaches enable a more comprehensive analysis of the tumor and its microenvironment compared to patch-level methods. Another common form of histopathology data is tissue microarrays (TMAs) data, where pathologists select a small circular representative region from multiple tumor blocks, which are then mounted on a single slide. This technique allows simultaneous imaging of samples from multiple patients, making the analysis more efficient and facilitating comparisons across different cases.

In the last few years, almost one paper on AI for digital pathology has been published every day highlighting its potential in this area. This is driven by several factors including data availability, technological advances, and increased interest by clinicians. With a large number of published papers, it is crucial to have an effective approach to evaluate existing deep learning approaches, their performance, and factors predictive of their success.

In this work, we built the AI in Histopathology Explorer (HistoPathExpo: www.histopathexpo.ai) as an online resource for interactive exploration of deep learning methods and their applications to different cancers and various clinical tasks in histopathology. We developed various tools within the HistoPathExpo that allow advanced analysis of our data. Our work enables researchers to gain valuable insights and performs a comprehensive analysis of published articles on AI in histopathology working towards accelerating the development of digital pathology applications and facilitating the creation of gold standards in this area. We focused on deep learning methods due to their demonstrated performance over conventional machine learning methodologies. Our goals are to allow researchers and decision makers to 1) identify and evaluate relevant studies and deep learning approaches that represent the current state-of-the-art for various pathological applications, 2) determine factors contributing to the enhanced performance of deep learning models, and 3) gain a deeper understanding of both challenges and opportunities for improvements to facilitate adoption and translation of these applications in the clinic.

## Results

**Fig. 1:**
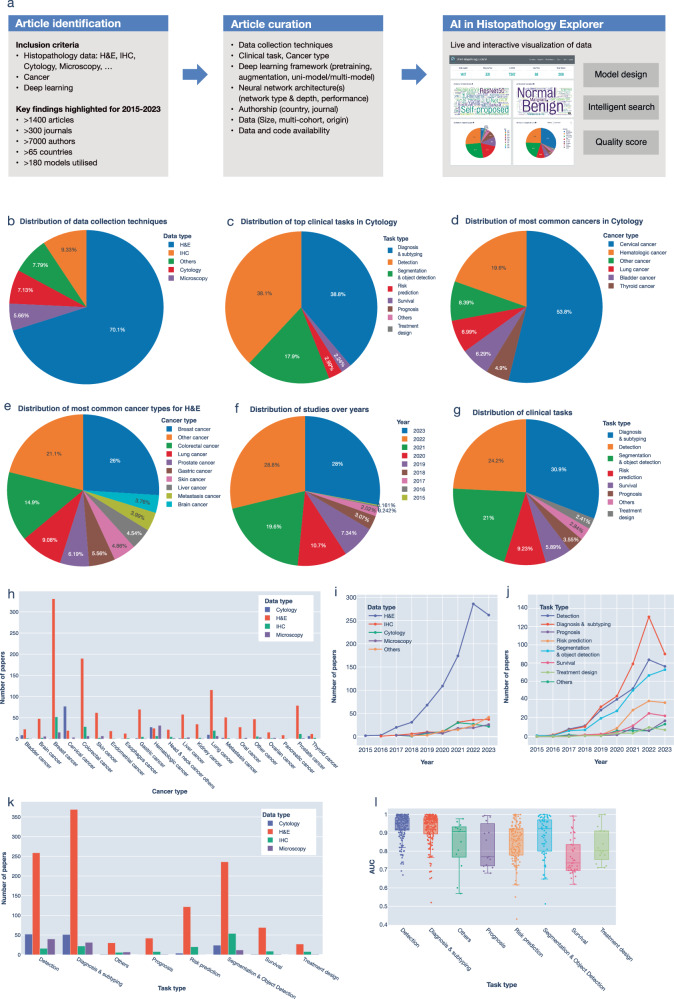
Summary of reviewed papers. **a** Workflow of the study. **b-g** Pie charts showing the distribution of papers across various data collection technologies (**b**), clinical tasks addressed in papers using cytological data (**c**), cancer types investigated using cytological data (**d**), cancer types investigated using H&E data (**e**), year of publication (**f**), and clinical tasks addressed (**g**). **h** Frequency of papers by cancer type, and data collection technique. **i** Trends in the number of studies using different data collection methods over the years. **j** Trends in the number of studies aimed at different clinical tasks over the years. **k** Number of papers by task, and data collection technique. **l** Model performance based on AUC across various clinical tasks.

We developed an online and real-time dashboard, HistoPathExplorer, to analyze the performance of published deep learning methods applied to histopathological data (Fig. 1a). This dashboard allows users to visualize and explore these AI applications across various cancer types, clinical tasks, neural network models, and datasets, providing an interactive platform for detailed analysis (Supplementary Fig. 1a, b). To this end, we curated the performance and methods from over 1400 published studies on deep learning applications in histopathology, reporting here the results from the period between 2015 and 2023 (Methods). We considered various cell imaging techniques due to the increased interest in these technologies and their amenability for clinical translation including Haematoxylin and Eosin (H&E), which consisted of 70% of studies, immunohistochemistry (IHC) and cytology (Fig. 1b–e, h, and Supplementary Table 1). Over the last few years, the number of research articles applying deep learning techniques to histopathological images has almost quadrupled, rising from 91 in 2019 to 357 in 2022, and maintaining a high level of 347 studies in 2023 (Fig. 1f). The most significant growth was observed in studies aimed at H&E images with a tenfold increase accounting for 70% of studies (Fig. 1i). Such rapid growth highlights a new era in digital pathology.

### AI applications and emerging trends in histopathology

Diagnosis and detection were the most common target tasks for deep learning models (30.9% and 24.2%, Fig. 1g, j, Supplementary Table 2). This is consistent across data types except for IHC (Fig. 1k). They also achieved the highest Area Under the Curve (AUC) of 96% (Fig. 1l). Examples of diagnosis tasks include tumor grading and cancer subtyping such as HER2+, ER+, PR+, or triple-negative tumors in breast cancer^10–12^, or sarcomatoid versus epithelioid tumors in mesothelioma^13^. The interest in diagnosis surged in 2022 with a 1.7-fold increase compared to 2021 and a 3-fold increase as compared to 2020 (Fig. 1j).

Segmentation and object detection was the third most popular task (21%, Fig. 1g), with almost a 3-fold increase between 2018 and 2022 (Fig. 1j). This was the most common task for models trained on IHC images (Supplementary Fig. 1c). Segmentation models can be trained to detect or segment different regions such as tumor, mitotic cells, Ki67+ cells, or various immune cell types^14–17^. In this category, we also included studies that might not provide direct clinical value but rather feed into other clinical tasks or another model such as graph neural networks. These include segmenting cells or tissue structures such as glands, and abnormal or tumor regions. For instance, Silva-Rodríguez et al. proposed WeGleNet that performs Gleason grading and extended it to segment regions representative of the various tumor grades^18^. Such an extension can result in more explainable models that can assist pathologists in confirming their diagnosis.

Risk prediction tasks (9.2%, Fig. 1g) included predicting various risk factors associated with prognosis or diagnosis. The number of studies aimed at risk prediction in 2023 is nearly four times higher than those published in 2020 (Fig. 1j). These studies include predicting genetic mutations predictive of risk including FLT3 and CEBPA mutations in Acute Myeloid Leukaemia^19^; p53 mutations in prostate and ulcerative colitis-associated cancer^20,21^; BRAF mutation in melanoma, bladder, colorectal or thyroid cancers^22–25^; or FGFR mutations in bladder cancer^26^. An interesting study by Kather et al. trained one model to predict various key mutations in different cancers using pan-cancer data^27^. Combining predicted risk scores from different AI models, could be an effective strategy to optimize patient treatment in the future^28^.

Survival and treatment design were defined as separate prognostic tasks (5.9% and 2.4%, Fig. 1g). For instance, Wang et al. proposed the Surformer model that combines multi-head self and cross-attention modules for predicting survival in different cancer types^29^. Other studies aimed at treatment design mainly focused on response to treatment, such as response to neoadjuvant chemotherapy in triple-negative breast cancer^30^, or response to immune checkpoint inhibitors in advanced melanoma^31^ which has the potential to support clinical trial design^32^. Given the large variability in patient treatment regimens, studies aimed at survival and treatment design had the lowest AUC of 80% (Fig. 1l). It would be important in the future to develop models that are robust to variations in treatment regimens across different countries and healthcare systems.

### Cancer-specific analysis of AI applications

**Fig. 2:**
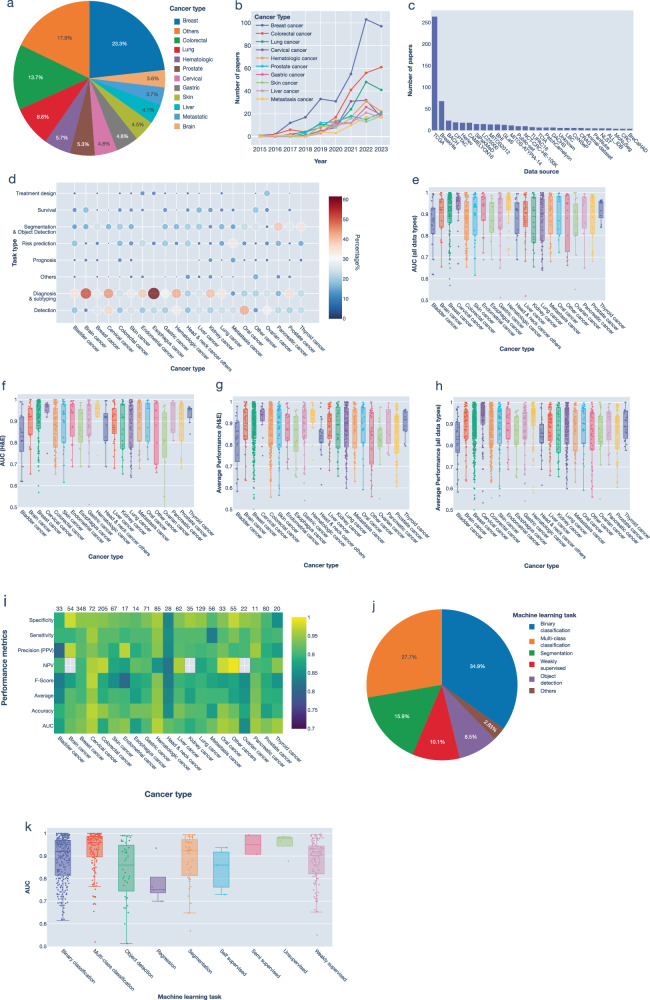
Summary of performance metrics by cancer type. **a** Pie chart showing the distribution of studies by most investigated cancer types. **b** Number of studies published per year for the most investigated cancer types. **c** Number of papers utilizing various publicly available datasets based on the number of publications. **d** Heatmap of the distribution of clinical tasks across different cancer types where the number of papers is normalized to the total number of studies per cancer type. **e-h** Box plots of various performance metrics by cancer type based on AUC for all data collection techniques (**e**), AUC for H&E data (**f**), average performance for H&E data only (**g**), and average performance for all data collection techniques (**h**). **i** Heatmap showing the median value of various performance metrics for each cancer type. The total number of papers included in the heatmap for each cancer type is shown above the heatmap. **j** Distribution of papers by machine learning tasks. **k** Box plot of model performance based on AUC across various machine learning tasks. AUC Area-Under-Curve, PPV Positive Predictive Value, NPV Negative Predictive Value.

Most AI studies were aimed at breast cancer (23.2%), followed by colorectal cancer (13.7%) and lung cancer (8.6%) (Fig. 2a). These are also the most common cancers worldwide^33^ and achieved the highest increase in the number of studies over the years (Fig. 2b and Supplementary Fig. 2a). Several public datasets, including those released as part of machine learning challenges, were available for these cancers such as the breast cancer datasets BreakHis^34^, and BACH^35^ (Fig. 2c). Breast cancer was also associated with the highest number of machine learning challenges (14/26) (Supplementary Tables 3, 4). This highlights the importance of publicly available data in driving innovation in AI applications.

The emphasis on clinical tasks differed among various cancers. For example, diagnosis and subtyping was the most common task for esophageal (61%) and brain cancers (53%) (Fig. 2d and Supplementary Fig. 2c). Detection task was more common in cancers that are easier to sample from, such as oral (42%), cervical (35%) and haematological cancers (32%). Segmentation was the most common task in pancreatic (38%), thyroid (33%) and prostate cancers (28%). For survival tasks, brain and liver cancers were the most common followed by kidney cancer. Less than 3% of studies explicitly mentioned treatment design, and these were aimed at breast, lung, colorectal, and ovarian cancers (Supplementary Fig. 2b). These results could indicate clinical needs as well as the accessibility of samples are critical factors in advancing computational pathology.

In terms of performance, we observe that models trained on ovarian cancer data have the lowest AUC (AUC of 0.85, *n* = 22), followed by bladder cancer (AUC of 0.87, *n* = 14) and esophageal cancer (AUC of 0.87, *n* = 33) across different data collection techniques (Fig. 2e). Ovarian cancer also performed the worst when considering H&E data only (Fig. 2f). To get an approximation of the general performance, we calculated the average of all performance metrics for each study (Methods). The average performance values revealed that while studies of head and neck cancers (except oral cancers) achieved a median AUC of 0.92, they had the lowest performance mean index of 0.84 due to their low specificity and sensitivity (Fig. 2g–i). Our findings highlight potential opportunities for enhancing the performance of digital tools across various cancer types.

### Performance evaluation of deep learning architectures

Most tasks were defined as classification tasks where the model aims to predict a binary class (35%) or multiple classes (28%, Fig. 2j). The models used in these tasks achieved the highest performance across all machine learning tasks (Fig. 2k). We also defined a distinct category for weakly supervised approaches where only image-level labels were utilised (10%, Fig. 2j). Weakly supervised approaches showed slightly lower performance, as anticipated due to the difficulty of accurately localizing relevant regions within the image, especially since it may contain heterogeneous or multiple objects with varying characteristics or features (Fig. 2k). Techniques that can learn from unlabelled or limited amounts of labelled data, like semi-supervised and self-supervised approaches^36,37^, are proving to be promising with an average AUC of 90%.

**Fig. 3:**
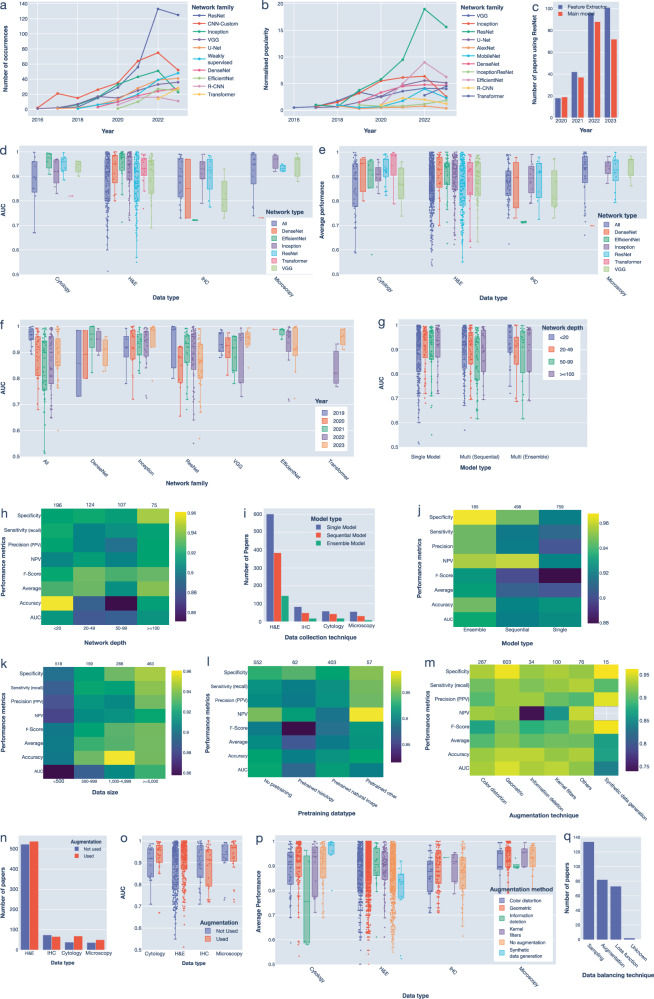
Summary of performance based on different model types and design factors. **a-b** Popularity of the most used networks over the years based on: the absolute number of studies (**a**), and on normalized popularity (**b**). CNN (Convolutional Neural Network), where CNN-Custom includes all bespoke architectures proposed. **c** Use of ResNet networks as a feature extractor model, and as main, or part of the main model from 2020 to 2023. The two categories may overlap. **d** AUC of popular network models by various data collection techniques where only approaches using a single-model were considered. **e** Average performance of popular network models by various data collection techniques. **f** AUC values of popular network types over 5 years (2019 to 2023). **g** Boxplot of AUC values of single, sequential and ensemble models when different number of layers were utilized. **h** Heatmaps showing the median value of various performance metrics for different network-depth ranges. **i** Number of papers using single, sequential, and ensemble models across various data collection techniques. **j-m** Heatmaps showing the median value of various performance metrics for different model types (**j**), dataset sizes (**k**), pretraining data types for H&E data (**l**), and image augmentation techniques (**m**). **n** Number of papers using data augmentation across various data collection techniques. **o** Box plot of AUC across different data collection techniques based on the use of data augmentation. **p** Average performance of different augmentation methods across various data collection techniques. **q** Bar chart showing the frequency of papers using different data balancing techniques for classification tasks.

The most used deep learning models were ResNet (AUC of 88%, introduced in 2016)^38^, Inception (AUC of 95%, 2015)^39^, VGG (AUC of 92%, 2014)^40^, EfficientNet (AUC of 93%, 2019)^41^, and DenseNet (AUC of 91%, 2017)^42^ (Fig. 3a, b, Supplementary Fig. 3a, b). These are convolutional neural networks (CNNs) that are well-suited for fully supervised classification tasks. Most of these networks appeared earlier in the Computer Vision field (Supplementary Fig. 1d and Supplementary Table 5). When considering their performance in studies that used single models, we observed high variability in network performance across data collection techniques (Fig. 3d, e). For example, ResNet demonstrated the highest AUC when applied to cytology data, but lower AUC for H&E and microscopy images that might benefit from slide-based analysis (Fig. 3d). ResNet has also been widely used as a feature extractor for further processing by other models. (Fig. 3c). Interestingly, EfficientNet and DenseNet had the best performance consistently across different data types, which explains their popularity (Fig. 3b and Methods). Notably, the performance did not necessarily improve over the years except for VGG- and transformer-based architectures which could be due to increased complexity and a higher number of studies (Fig. 3f). In contrast, transformers performance has significantly improved between 2022 and 2023, highlighting their potential for further advancement.

Network depth correlated with a better performance especially when more than 100 layers were utilized (Fig. 3g, h and Supplementary Fig. 3f). Half of the studies employed multiple models, with 37% using them sequentially and 13% using them concurrently (i.e., ensemble models). Furthermore, 78% of the studies involving multiple models focused on H&E images, which are typically much larger than images obtained through other methods (Fig. 3i). Studies that employ ensemble models have higher performances based on various measures including AUC, specificity, and sensitivity compared to papers that employed one model or multiple models sequentially (Fig. 3j, one-way ANOVA *p*-value = 0.0097). These results suggest that combining predictions from different models can be an effective strategy for enhancing performance.

### Impact of study design and implementation on performance

Dataset size correlated with a better average model performance (Fig. 3k, Spearman correlation coefficient = 0.171, *p-*value = 3.6e^−10^, Supplementary Fig. 3c–e). Surprisingly, studies employing single model architectures were more sensitive to dataset size compared to studies employing multiplemodels sequentially (Supplementary Fig. 3d). Pretraining on other datasets, such as ImageNet or biomedical data, was often used to mitigate overfitting in small datasets (48% of studies, Fig. 3l). We did not observe performance advantage when the models were pretrained on histopathological images, except for specificity. This could be due to the large size of natural image datasets.

Data augmentation, used in 53% of studies, was another popular strategy for addressing small dataset size and reducing overfitting by effectively increasing the number of examples available to deep learning models (Fig. 3m–p). Augmentation improved AUC in cytology and H&E studies, confirming its importance (Fig. 3o). The most commonly used augmentation method is the geometric-based approach which also performs significantly better than other augmentation methods (Fig. 3p, Supplementary Fig. 3g–j). These methods include rotating^43^, flipping^44^, scaling^45^, elastic distortion^46^, or affine transformation^47^ of an image (Supplementary Table 6). On the other hand, studies that used synthetic data generation by generative models have the lowest performance except for specificity metrics (Fig. 3m, Supplementary Fig. 3g-i). It remains unclear whether this reduced performance is due to the limited amount of data or suggests that synthetic data generated by current methods may not be as effective as simpler augmentation techniques.

Augmentation was also used to tackle imbalance in classes which was utilized in 8% of studies. Other strategies for tackling this include various sampling strategies (10%), and modified loss function (5%) which adjusts the weights for a given class (Fig. 3q, Supplementary Table 7). Such approaches are often associated with increased performance across different data collection techniques (Supplementary Fig. 3k).

### Approaches toward trustworthy AI

**Fig. 4:**
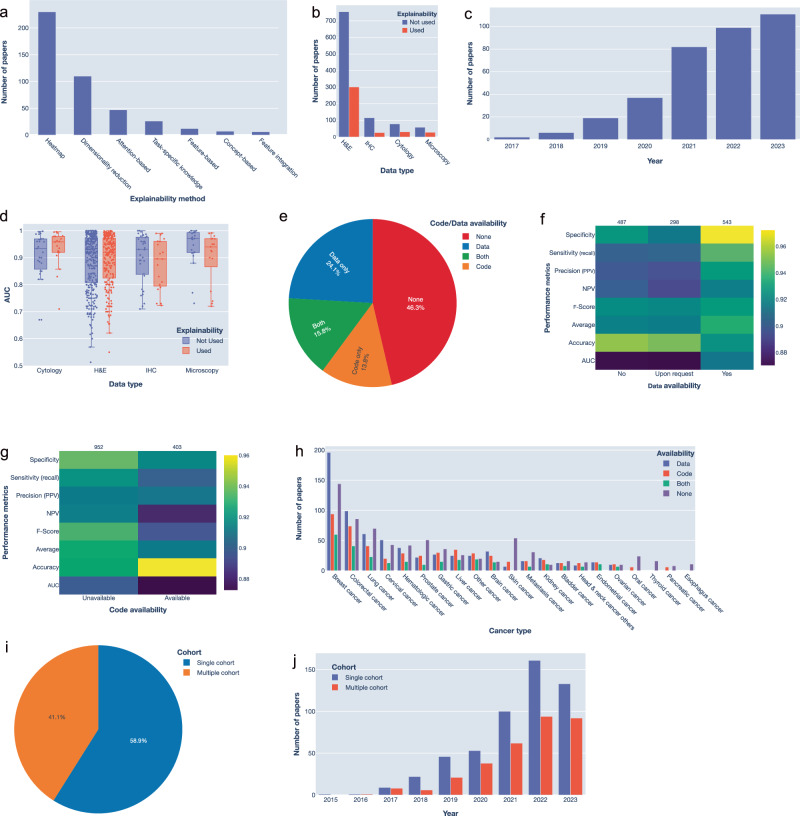
Analysis of explainability and reproducibility in the reviewed studies. **a** Number of publications employing various techniques for model explainability. **b** Number of publications employing explainability across different data collection techniques. **c** Use of explainability methods from 2017 to 2023. **d** AUC values of models employing explainability across different data collection techniques. **e** Percentage of studies with available data and code, or both. **f-g** Heatmap of the median of various performance metrics based on: data availability (**f**), or code availability (**g**). **h** Number of publications with available data, or code for each cancer type. **i** Percentage of papers using a single-cohort or a multi-cohort dataset. **j** Change in the number of publications using single- versus multi-cohort datasets over the years.

Explainability approaches aim to ensure that the model effectively learns relevant pathological signatures, thereby increasing the confidence in the accuracy of its predictions. Only 28% of studies utilized explainability techniques whereas 77.4% of those were published after 2020 (Fig. 4a–c, Supplementary Table 8). Explainability was associated with a significantly higher median AUC in cytology but did not result in a significant difference in H&E studies (Fig. 4d). The most common approach for explaining model prediction is Class Activation Maps method (CAMs)^48^, and its variations such as Grad-CAM and Score-CAM^49,50^ which highlight relevant image regions in a heatmap (53%, Fig. 4a). Dimensionality reduction such as Principal Component Analysis (PCA), t-Distributed Stochastic Neighbour Embedding (t-SNE) or Uniform Manifold Approximation and Projection (UMAP) is another common method that allows inspecting the similarities between samples from different classes to gain a better understanding of the learned predictions. For instance, Liang et al. used t-SNE to show that images associated with lymph node metastases were clustered together in the latent space, proving that the model identified relevant features^51^. Other approaches include attention mechanisms which are inherently used in transformers and concept-based identification. We propose that combinations of various explainability methods might be needed for a better understanding of complex deep learning methods.

Another critical factor for trustworthy AI is reproducibility. While data and code availability are essential for this purpose, we found that 46.3% of papers do not have either available (Fig. 4e–h). Certainly, large consortium efforts, such as TCGA, provided valuable datasets for benchmarking and assessing reproducibility. For example, data from TCGA has been utilized in 19.5% of studies (Fig. 2c). Surprisingly, the number of studies with data available is higher than those with code available (24.1% versus 13.8%, Fig. 4e and Supplementary Fig. 4a, b). Importantly, the availability of data is associated with significantly better performance, potentially due to the consistent benchmark providing a stable reference point for method development (Fig. 4f, *p*-value = 0.002 based on one-way ANOVA test). Nearly 40% of the studies tested their methods on multiple cohorts, but this did not necessarily increase in recent years (Fig. 4i, j). These findings highlight the importance of data, and code sharing for fostering reproducibility, and advancing method development.

### A comparative analysis of foundation models in histopathology

**Fig. 5:**
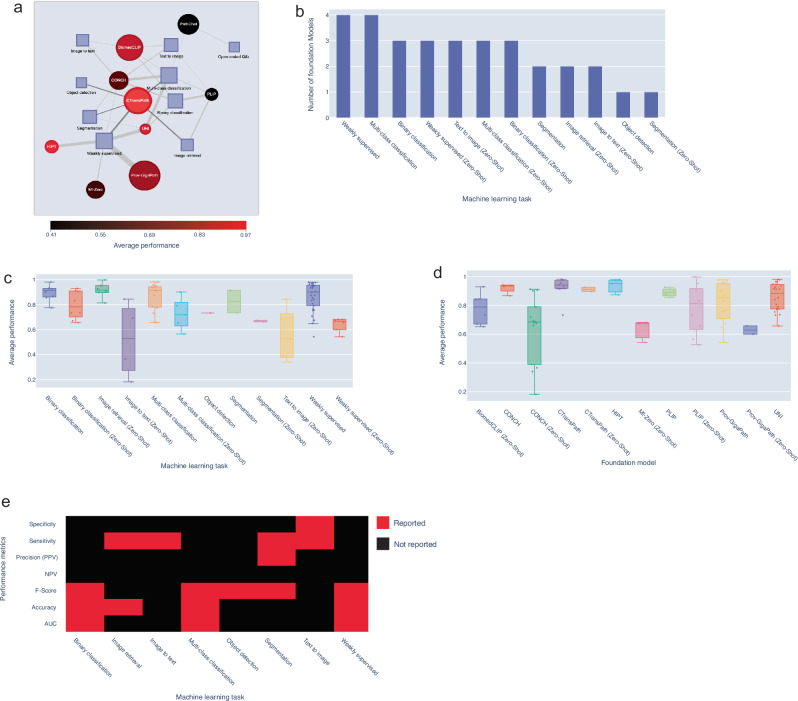
Evaluation of foundation models. **a** Network representation of machine learning tasks investigated by different foundation models. The weights of the edges between models and tasks represent the number of tasks investigated. The average performance of the foundation model is indicated by node colour. Node size indicates the connectivity of the node. **b** Number of foundation models investigating various machine learning tasks. **c-d** Average performance across various machine learning tasks (**c**) and various foundation models (**d**). **e** Reported metrics investigated by different machine learning tasks.

Several foundation models have been introduced recently, each trained on extensive datasets to serve as versatile tools for a range of machine learning applications, including captioning, classification, and segmentation. These models can be used directly, without additional fine-tuning (zero-shot), or can be further finetuned for specific tasks. We focus here on models evaluated on clinical tasks. CONCH^52^, CTransPath^53^, PLIP^54^ and BiomedCLIP^55^ employed contrastive self-supervised learning that learns to distinguish similar and dissimilar images and/or text pairs. On the other hand, image masking was used in UNI^56^ while HIPT^57^ and MI-Zero^58^ models used traditional supervised learning. To evaluate foundation model performance, we curated the performance and datasets for each task separately (Methods). We found that all models were validated based on weakly supervised, binary or multi-class classification tasks (Fig. 5a, b). CONCH^52^, UNI^56^ and CTransPath^53^ were also evaluated on segmentation tasks such as gland segmentation, and mitosis detection. Moreover, models such as CONCH^52^, PLIP^54^ and BiomedCLIP^55^ were trained to perform image-to-caption and caption-to-image tasks. Foundation models performed well in various finetuned tasks where binary classification and weakly supervised learning achieved the highest average results, exceeding 90% (Fig. 5c). For zero-shot learning tasks, the average performance ranged from 52.7–73.2%, indicating the importance of finetuning (Fig. 5c, d). We note that the limited number of reported performance metrics, as seen with models like HIPT^57^, ProvGigaPath^59^, and PathChat^60^, restricts a comprehensive evaluation of their capabilities (Fig. 5e). Addressing these gaps presents an opportunity to further refine foundation models, enhancing their versatility and robustness for diverse applications in machine learning.

### Use-cases and intelligent tools for data-informed model design

**Fig. 6:**
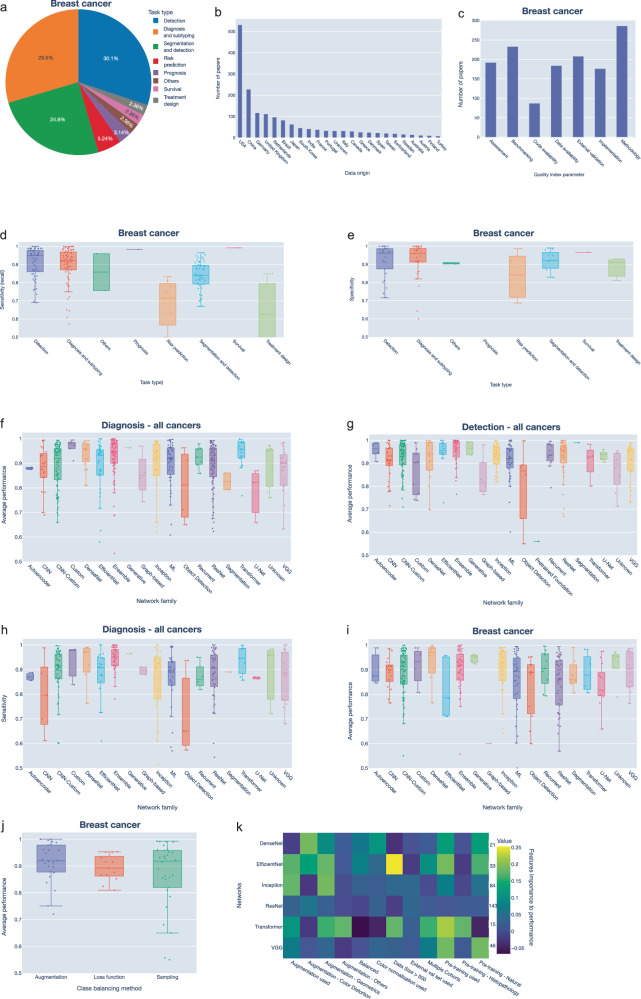
Example use of HistoPathExplorer using filtering functionality. **a-c** From the Summary page, the user can obtain cancer-specific plots by filtering for breast cancer. Shown are the ‘Distribution of papers by task’ plot (**a**), ‘Distribution of papers by data origin’ plot (**b**) and ‘Distribution of papers by quality index parameters’ plot (**c**). **d-e** Plots from the Task page under Performance menu showing ‘Performance by clinical task’ when ‘Breast cancer’ with (**d**) ‘Sensitivity’ or (**e**) ‘Specificity’ are selected. **f-i** Results from the ‘Performance by network family’ plot from the Models page under Performance menu, selecting (**f**) ‘Diagnosis and subtyping’ task, (**g**) ‘Detection’, (**h**) ‘Diagnosis and subtyping’ task with ‘Sensitivity’ metric, or (**i**) ‘Breast cancer’. **j** Results from the ‘Performance by class balancing method’ plot on the Implementation page under Performance menu with breast cancer selected. **k** Importance of various features to the average performance of top-used models based on ReliefF feature selection algorithm in the Feature Ranking tool.

**Fig. 7:**
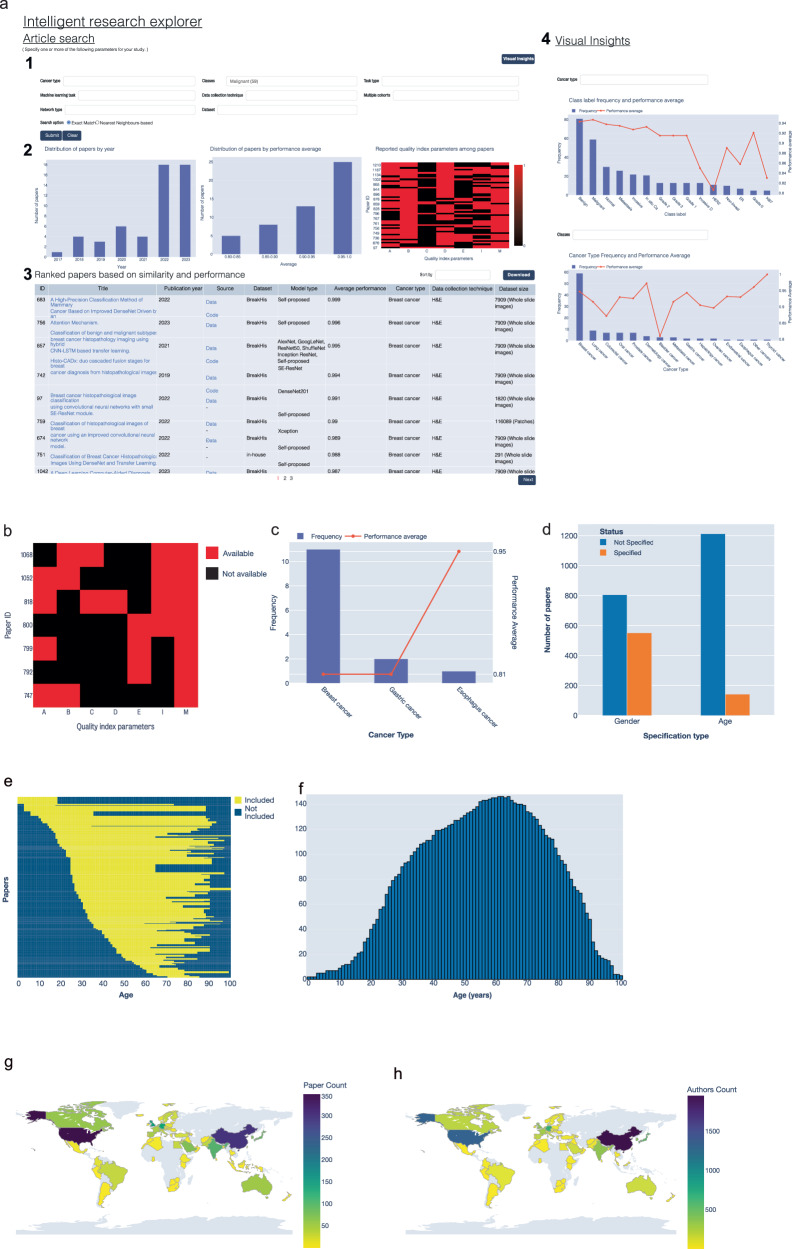
Demonstration of Intelligent Explorer functionality. **a** A screenshot of the Intelligent Explorer. (1) The search articles panel allows searching for articles based on specific properties such as cancer type, implementation details, and data utilized. (2) Given a certain user query a visual summary of performance, publication dates and quality indicators is displayed. (3) A list of articles and key features that is also available for download. (4) The Visual Analytics panel allows quick investigation of studies performed on a certain cancer or for a certain clinical class. **b** Quality indicators for papers predicting HER2 status from H&E images. A: Assessment, B: Benchmarking, C: Code, D: Data, E: External validation, I: Implementation details, M: Methodology. **c** Number of studies investigating HER2 in different cancer types. **d** Number of papers specifying the gender, and age investigated in their studies. Studies investigating gender-specific cancers such as prostate, and gynaecological cancers were assumed to implicitly specify gender. **e** Age range distribution across papers, with each yellow line representing the age range for a specific study. **f** Frequency of age range used in datasets. **g** World heatmap highlighting the number of papers published by each country based on the senior author country of affiliation. **h** World heatmap highlighting affiliation countries of all authors of reviewed papers.

**Fig. 8:**
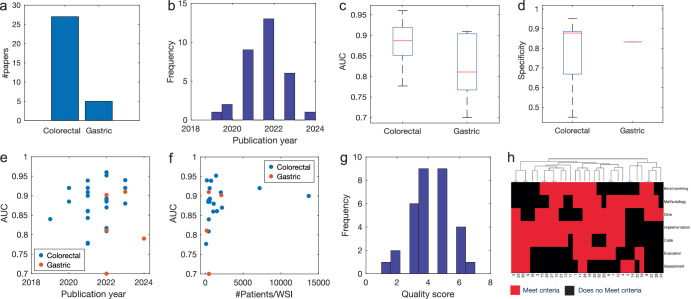
A use-case based on Microsatellite Instability (MSI) detection. **a-b** The number of published studies investigating MSI in different cancer types (**a**) and over the last few years (**b**). **c-f** Performance of studies aimed at MSI prediction based on AUC (**c**), Specificity (**d**), and its correlation with publication year (**e**) and the number of patients or whole slide images (**f**). **g** Distribution of quality scores for MSI publications. **h** Clustering of the values for the individual quality indicators for papers in Supplementary Table 10.

We provided various functionalities in HistoPathExplorer, to support a wide range of users in obtaining insights, evaluating new tools, understanding deep learning capabilities as they evolve, and developing deep learning methodologies. The web pages under the Analysis, and Performance menus allow interactions with the data to filter for specific cancers or tasks and create customised figures based on the latest articles. The plots on the dashboard are clickable, allowing users to interact with any component to access associated papers and perform a more thorough investigation of certain methods or results. Moreover, we created several intelligent tools within HistoPathExplorer under the Tools page to allow more advanced search of relevant studies: (1) the Intelligent Explorer, a tool that retrieves the details of the most relevant approaches based on the user input and provides graphs summarising the performance and various quality indicators (Methods); (2) a Feature ranking tool to determine the impact of various choices on model performance. Figures 6–8 highlight the utility of these different functionalities.

#### Landscaping and high-level assessment of histopathology literature in breast cancer

HistoPathExplorer allows users to explore broad trends, providing key insights into how deep learning models perform across different cancer types and clinical tasks. To illustrate the utility of the developed dashboard, consider an engineer or clinician aiming to develop a robust deep learning method for diagnosing breast cancer. They can use the dashboard to examine the landscape of studies focused on breast cancer and understand existing methods. From the Summary page, they can view the distribution of different clinical tasks and identify that ‘Detection’ was investigated in 30% of breast cancer studies, while ‘Diagnosis’ was investigated in 29.6%. They can also identify gaps in research with only 5.24% of studies focusing on risk prediction and 2.36% on treatment design, suggesting areas for innovation (Fig. 6a). The ‘Distribution of papers by data origin’ plot reveals a good representation of breast cancer studies from different countries including USA, China, Germany, UK, the Netherlands and Brazil (Fig. 6b). Plots of quality indicators show that only 48.7% of the studies reported three or more metrics as indicated by the Assessment feature (Fig. 6c). A list of these studies or those meeting other indicators for breast cancer can be obtained by clicking on the relevant bars in the plot.

Users can evaluate reported performance through pages in the Performance menu. On the Task page, filtering for breast cancer reveals that studies aimed at either ‘Detection’ or ‘Diagnosis and subtyping’ achieved a median sensitivity above 93% and specificity of 97% (Fig. 6d, e). However, ‘Detection’ studies show a wider variability in specificity with an interquartile range of 10% versus an interquartile range of 6% for ‘Diagnosis and subtyping’ suggesting potential for improvements. Users can further assess the performance of specific deep learning models by filtering based on cancer type or task type within the Models page. For example, when considering studies aimed only at ‘Diagnosis and subtyping’, studies employing EfficientNet, DenseNet and Transformers are among the models with the highest average performance metrics (Fig. 6f). A similar trend is observed in ‘Detection’ studies, indicating consistent model performance (Fig. 6g). Users interested in developing methods with a high sensitivity might focus on DenseNet and Transformers, which stand out when selecting this metric (Fig. 6h). Users can also view the average performance of these models specifically for breast cancer which supports the potential of DenseNet and Transformers (Fig. 6i).

The Implementation page allows users to assess the impact of various factors on the performance. For example, the user can directly compare the performance of different data preprocessing and augmentation strategies, such as class balancing or synthetic data generation. They can identify that studies employing class balancing through augmentation appear to have better performance in breast cancer (Fig. 6j). The user can obtain further insights on the use of these models and various implementation details from the Models page under the Analysis menu. For instance, they can determine the various explainability approaches in histopathology. To find recent methods that incorporate Task-specific knowledge, users can click on the relevant bar for an updated list of studies. They can also explore studies using the Geometric augmentation technique by clicking on it in the ‘Distribution of papers by augmentation technique’ plot to filter and search through the results. The Terminology page offers further details on the methods in each category. Together, these different pages allow users to quickly identify, and access the latest studies to evaluate diverse design considerations.

#### Feature ranking tool

To provide a more systematic evaluation of the impact of various model features, we created a ranking tool to evaluate the significance of different implementation aspects for the top-performing models (Methods). For instance, from the ‘Design assistant’ page under the ‘Tools’ menu, the user can determine that using pretraining on natural images was most important for EfficientNet, VGG, and DenseNet (Fig. 6k). While pretraining on histopathological data was more important for transformer-based models. Moreover, Dataset size was the most important for EfficientNet and Transformers. These tools equip researchers with a data-driven approach to crafting their deep learning strategies, highlighting the most critical factors to consider during model development.

#### Informed model design using the Intelligent Explorer

The Intelligent Explorer enables detailed searches to identify relevant studies and assess dataset and model quality, metadata, and other key characteristics in one place (Fig. 7a). For example, through the Tools menu, an engineer can access the Visual Insights panel within the Intelligent Explorer page to view the number and average performance of papers across identified clinical classes. (Fig. 7a). The engineer can determine that the most frequently studied classes in breast cancer include tasks such as benign, malignant, HER2, ER, and Ki67 status, metastasis, various cancer grades, and specific subtypes like invasive ductal carcinoma. Malignant class was investigated in 59 studies with an average performance of 93%. By selecting the Malignant class in the left search panel, he can view visual summaries of publication dates, performance and quality indicators, as well as the details of individual studies. The user can also easily identify from the list of papers that many of these studies are associated with BreakHis and BACH datasets. Models with the best-reported performance include Xception, DenseNet, SE-ResNet, and ViT, with average performance exceeding 98%. This supports the previous observations on the potential of DenseNet and Transformers based on a larger number of studies. The quality indicators heatmap allows users to select studies with the required details. For example, they can focus on the four studies with publicly available code (Fig. 7a). He may also use the dashboard to find usable datasets from the Summary page, where he can choose studies with data from different countries of origin as well as diverse demographic groups. This allows creating a more comprehensive dataset, toward enhancing the model generalizability and reliability across various patient populations (Fig. 6b). The engineer can also visualize and understand the complex relationships between clinical classification problems, clinical tasks, cancer types, datasets, and deep learning models using the Network tool (Supplementary Fig. 4d). These functionalities and insights assist engineers to effectively design their study and develop strategies for selecting models and testing datasets.

#### Example application of the dashboard in clinical research

A clinician interested in the HER2-positive breast cancer, can use the Intelligent Explorer to find studies aimed at predicting HER2 status in breast cancer. This shows that seven studies aimed at predicting HER2 from H&E images with only four studies having a quality indicator score greater than three (Fig. 7b and Supplementary Table 9). Among these, HAHNet, which utilised publicly available data and employed an InceptionV3 backbone, has the best average performance of 94.5%^61^. Another model by Bae et al. reports an average performance of 85.8%^12^. These metrics could motivate the creation of a clinical tool to detect HER2 from H&E images. Publicly available data and code from these studies can facilitate the development and benchmarking of a digital biomarker for HER2 detection. Given the high performance of Inception-based architecture, the feature ranking tool can be used to further explore key implementation details, revealing that data augmentation and dataset size are top contributors to its performance (Fig. 6k). The clinician can also check if HER2 has been studied in other cancers from the Visual Insights panel revealing its application in gastric and esophageal cancers^62,63^ (Fig. 7c). This framework equips engineers, scientists, and clinicians with the tools to efficiently explore and utilize AI methodologies in their specific medical research contexts.

#### Use case for decision makers and regulators

HisotPathExpo dashboard can be valuable for decision-makers such as regulators and policymakers by providing a quick overview of related studies^64^. For example, the Intelligent Explorer can support a regulator in evaluating a new AI tool for predicting microsatellite instability (MSI) in different cancers. MSI is a genotypic signature caused by a deficiency in DNA mismatch repair^65^. It serves as an important biomarker and a risk factor in several cancers including gastric, colorectal, lung, and endometrial cancers. Its detection helps to match patients with certain treatments, particularly in colorectal cancer where patients with high MSI scores are more sensitive to immunotherapy^65^. MSI can also be a risk factor for patients with Lynch syndrome^66^. The pioneering work of the Kather group has led to the first clinically approved slide-based AI tool for MSI detection in colorectal cancer patients in Europe^65–68^.

Using the Intelligent Explorer, a regulator would be able to instantly view 32 published articles aimed at MSI prediction using H&E images when selecting Microsatellite Instability (Supplementary Table 10). She can download associated data for her own analysis. Various insights can be generated showing that MSI prediction was applied to colorectal cancer in 27 studies and gastric cancer in 5 studies and the number of publications per year (Fig. 8a, b). Reported AUC values range from 0.7 to 0.96 (median: 0.88), with specificity ranging from 0.45 to 0.95 (median: 0.867) in colorectal cancer, though specificity was only available in 9 studies (Fig. 8c, d and Supplementary Table 10). Similar AUC ranges (0.7–0.91) were observed in gastric cancer, with a median AUC of 0.81, but data on other metrics were limited. Performance trends over time, shown alongside publication year and dataset size, reveal that increased research interest has driven performance improvements (Fig. 8e, f). The reported performance metrics offer a comparative benchmark for the regulator to assess whether the new tool performance aligns with published literature, especially those based on publicly available datasets. The quality indicators can provide further context helping the regulator to decide which studies to consider. For example, she might focus on studies that report at least three performance metrics (Assessment quality criterion, Fig. 8g, h). By carefully examining these studies, regulators can identify critical questions and determine whether the new tool adheres to best practices in study design.

Most of the methods aimed at MSI detection were developed based on the TCGA data. The regulator can see other publicly available datasets and their geographic origin (Supplementary Table 10), upon which evaluation of additional data can be requested. For instance, if currently the tool was evaluated only on data from Europe and the US, she can ask for validations on Asian datasets such as PAIP2020 dataset originating from South Korea^69^, SCRUM-Japan GI-SCREEN dataset from Japan^70^, and a dataset from China^71^. The dashboard also highlights demographic diversity in studies and whether the age range of various studies was reported which, in the case of MSI, can be highly valuable for patients with hereditary diseases such as Lynch syndrome. The regulator can also identify from the dashboard which papers proposed an explainability component and check those papers in more detail to identify potential analyses for assessment of the trustworthiness of the tool. For example, Wanger et al., proposed a patch-specific MSI score and evaluated the presence of various cell types and tissue phenotypes such as mucosa, stromal, neoplastic, vessels, and lymphocytes^67^.

As a comparison to our work, a recent review of AI methods for detecting MSI from histopathological images reported the performance of 10 published methods in terms of AUCROC and the size of the dataset^72^. In contrast, our dashboard allowed searching more than 1400 articles providing instant access to a broader set of relevant articles along with key details of study design. It facilitated generating summary graphs and filtering based on various features. We outlined a range of scenarios where the dashboard can offer valuable insights for regulators, researchers, and clinicians, to comprehensively evaluate the landscape of a specific clinical problem.

### Identifying challenges and opportunities in digital pathology

Our analysis revealed that a significant challenge in this field is to develop standardized methods for evaluating the performance of different AI tools in healthcare. The choice of performance metrics depends on the specific clinical task, as different tasks could tolerate different types of errors. For example, in tumor detection, higher sensitivity, even at the expense of false positives, may be more desirable, while treatment selection might require greater emphasis on specificity. Our analysis revealed that sensitivity and accuracy are commonly used for detection, while AUC and sensitivity are more commonly reported for treatment design (Supplementary Fig. 4c). Furthermore, some tasks require different evaluation metrics. For instance, while the Dice score is commonly used for segmentation tasks, it does not explicitly account for object count or class relationships. In contrast, the concordance index is more appropriate when predicting survival outcomes. To facilitate the evaluation of AI tools for clinical use, it is crucial to establish standards that account for these diverse requirements.

Another challenge arises when evaluating the performance of tools that aim at performing multiple tasks, such as foundation models. Special attention must be taken to ensure that performance does not vary significantly across different cancer types or clinical tasks. This could arise due to distinct pathological features or higher variability exhibited in some cancers. To address this, careful stratification of the data and task complexity is essential when evaluating the models to minimize performance disparities. Incorporating continual learning techniques can further enhance model adaptability by allowing models to learn and improve incrementally as new data and tasks are introduced^73^. This approach helps maintain consistent performance across various clinical scenarios, ensuring that models remain robust and effective even if they encounter new cancer types or more complex tasks.

In addition to the complexities of evaluating AI models for multiple tasks, a significant challenge lies in ensuring broad applicability across diverse populations. This could promote health equity as histopathology offers a cost-effective and widely accessible tool. However, the lack of diverse representation in most existing studies, limits its ability to benefit patients from all backgrounds. Importantly, this is also a crucial factor for ensuring a fair and inclusive healthcare system. For example, we find that only 40% of studies specified gender and just 11% of studies specified the age range of the patients (Fig. 7d-f). Additionally, most datasets originated from the US (32%), and China (13%) as these countries were leading in the number of published articles (Fig. 7g, h). Federated learning offers a potential solution by enabling the evaluation of methods across diverse, decentralized datasets from multiple countries while safeguarding patient privacy and data security^74^. Synthetic data augmentation and adversarial debiasing^75^ are other potential approaches to address these limitations and develop equitable, inclusive digital pathology tools.

## Discussion

Here we performed the first comprehensive analysis of deep learning approaches in histopathology to support the development of AI applications, and promote their adoption across global healthcare systems. Our analysis highlighted key challenges for translating these AI solutions into clinical practice, and revealed interesting insights into the evolving AI landscape in histopathology. To accelerate these developments, we created HistoPathExplorer, a platform that allows users to search, download and submit newly identified papers. Most importantly, we developed novel intelligent tools that harness our analyses toward data-informed model design, offering actionable and relevant information for researchers, clinicians and decision makers.

One major challenge that emerged from our analysis is the multi-faceted nature of evaluating AI models and interpreting performance metrics. The variability in performance across different clinical tasks could be due to data availability, or difficulty of the task. For instance, diagnosis tasks are often associated with readily available labels based on clearly defined standards, while tasks such as response to treatment could be complicated by many confounding factors such as the treatment regimens, other health conditions, comorbidities, and side effects. Additionally, treatment and prognosis require follow-up data that could be more difficult to obtain. Our dashboard allows searching for best-performing papers given a specific clinical task, thereby facilitating more accurate comparisons.

Explainability and interpretability of predictions can be important aspects of model evaluation. Our results revealed that almost a third of the studies investigated the explainability of deep models to determine whether model predictions are associated with relevant pathological processes or clinically relevant regions in the image. However, these approaches often lack a systematic scoring of explainability. Moreover, explainability approaches could be contsrained by human perception and understanding of visual information, limiting the discovery of more complex quantitative patterns that might not be apparent to humans. This has been tackled in some studies by integrating domain knowledge and genomic features^76^. Additionally, more explainable models, such as graph networks, might not offer a performance advantage. This raises a debate about whether we should focus on building more accurate approaches or find a compromise between accuracy and explainability.

An important future direction is to ensure the fairness of the developed AI methods that deliver consistent performance across diverse populations and socioeconomic contexts, including underrepresented groups and individuals with rare cancers. While targeted collection of more diverse datasets is generally seen as the solution, a key challenge is ensuring that models retain their performance on the original population while adapting to new, diverse data. This phenomenon, known as catastrophic forgetting, can limit the model ability to generalize effectively when updated with additional data^77^. Few-shot learning that adapts to a new domain based on a few examples might be a more effective strategy in these cases^78^. However, this approach might not work well if there are inherent differences in the disease process in certain subgroups. Crowdsourcing data from social media has been proposed recently to create more diverse datasets, and has demonstrated success in predicting Gleason grade^79^. However, this raises ethical concerns regarding patient privacy, data validity, and traceability, which must be carefully addressed. Another emerging, and potentially more practical, approach involves developing auxiliary modules to address biases, through either preprocessing or postprocessing strategies^80^. Preprocessing strategies focus on extracting features, trained using representation learning, that are invariant to subgroups^81^. This can be achieved by training on large datasets, as seen in foundation models^82^. It is important to ensure that these approaches do not inadvertently introduce new biases into downstream model training. Postprocessing strategies, on the other hand, aim to correct model bias after training. For instance, calibration modules that adjust model weights for different subgroups have been explored to mitigate biases^83^. Another key consideration when training such models is that certain labels, such as overall survival and treatment response, may be more susceptible to bias, as they can be influenced by socioeconomic factors and access to quality healthcare. This highlights the need for tailored fairness strategies depending on the clinical task and dataset characteristics.

Looking ahead, we believe that the future of digital pathology lies in the integration of diverse models to address various aspects of clinical tasks. In addition to the actual performance, trustworthiness, and fairness of AI systems are other critical factors that need to be systematically evaluated to ensure that AI systems are reliable, and accurate. We propose that methods for evaluating explainability, and fairness can be designed as auxiliary models that can be used by third parties such as regulatory bodies. Healthcare providers and regulators must have the right tools to assess AI applications and consistently measure the efficacy, accuracy, and reliability of AI-based tools. Therefore, developing standards, and platforms for evaluating different method performance is crucial for ensuring patient safety, and optimal treatment plans.

## Methods

### Article inclusion criteria

Data collection technology, disease, and algorithm design were the primary criteria for article inclusion. We considered papers published from 2015 onwards, a year that marked a turning point with increased interest in this area. For a fair comparison, we have limited the results presented in this article to articles published up to 2023, except for foundation model studies. We included the following histopathological data collection techniques: H&E images, IHC, microscopy, multiplexed imaging, spectral imaging, and other cytological and histopathological techniques. We focused on studies utilizing deep learning algorithms, and histopathological datasets in cancer. Studies matching our inclusion criteria were obtained from PubMed by using key search terms including ‘cancer’, ‘histology’, ‘pathology’, ‘histopathology’, and ‘deep learning’. We also utilized a natural language processing approach (AI For Health portal)^84^ and snowballing was also used to ensure the comprehensiveness of our resource. Each paper was screened manually to determine its suitability for our study. This resulted in 1355 articles between 2015 and 2023. Papers using microscopy datasets or microscopy datasets in combination with other data collection technologies (e.g., CT or MRI scan images) were also considered. All papers were curated, and checked by 3 individuals.

### Article curation

We defined and extracted 160 variables to capture various aspects of the study design, and clinical tasks. These include task information, dataset information, algorithm design, and paper information. All variables were recorded in an Excel spreadsheet.

Task information includes cancer types, clinical tasks, and machine learning tasks. Each paper will have a subspecialty and a cancer type, and will have one clinical task assigned only (Supplementary Table 2). For example, a paper aimed at colorectal cancer subtyping will have ‘subspec_colorectal’, ‘spec_gi’ (short for gastrointestinal) and ‘task_diagnosis+subtyping’ as true. It should be noted that papers using pan-cancer data were recorded separately instead of marking all single cancer types. This is to ensure fair comparisons in performances when filtered by cancer types. Machine learning tasks were recorded as a categorical variable, with values selectable from binary, multi-class classification, segmentation, etc. If more than one task or cancers were evaluated in one paper, they would be annotated in separate rows.

Dataset information covers data countries of origin, number of patients, image type (e.g., WSIs, patches, TMAs), dataset size (including training, testing, validation), input image dimension (e.g., 224*224), data collection technology (e.g., H&E, IHC), dataset availability, code availability and link if public, class labels if the study aimed at a classification task, whether multiple cohorts and whether external validations were used, as well as the gender and age of a patient.

Algorithm design variables include preprocessing techniques, explainability methods, deep learning architectures, and performance evaluations. These methods were grouped into categories to enable effective analysis (Supplementary Table 6–8). Preprocessing techniques include data augmentation techniques, data balancing techniques, and pretraining data types (e.g., histopathology images, natural images, etc), dataset used for pretraining (e.g., TCGA, ImageNet). Variables describing deep learning architectures specify the network type, depths, explainability/interpretability methods used, whether benchmarking was used, feature extraction networks, and whether they employed multi-models. For network types, we also defined the general family if applicable (e.g., ResNet model family includes ResNet18, ResNet50, ResNet101, ResNet152, ResNeXt101, and SE-ResNet). Network depth is defined as the number of layers for the deepest model if more than one model is used. Additionally, an ‘algorithm pipeline’ variable is established to help illustrate the workflow (e.g., U-Net (foreground segmentation) -> ResNet50 (feature extractor) -> autoencoder -> MLP). Performance evaluation includes standard metrics – AUC, precision, specificity, Negative Predictive Value (NPV), sensitivity, F-score, accuracy, and concordance index (c-index) for survival tasks.

To define the quality index, providing some indications of the comprehensiveness of the reported methodology, we added a ‘methodology’ variable that indicates that the paper clearly specified the algorithm design and dataset information. Moreover, we defined ‘Implementation details’ to indicate whether the learning rate, optimizer, and loss functions have been clearly stated in the paper or through providing the code. These variables provide more insights into the method reliability and reproducibility.

Other extracted variables include authors, author affiliations, article types (e.g., journal, conference), journal name, journal countries, journal impact factor, and abstracts.

### Model average performance

The average performance is computed as the mean of common metrics such as AUC, specificity, sensitivity, F-score, and accuracy. Metrics that are aimed at specific tasks such as concordance index, correlation or Cohen’s kappa coefficient were not included as they may significantly deviate in their values from commonly recognized metrics.

### Foundation models curation

We added a separate web page for foundation models for comparison. We curated the performance for each clinical and machine learning task separately. We identified a few additional variables including the size of pretraining data, specifying the number of images the model trained on, and the use of zero-shot learning. We also added new machine learning categories to indicate if the model is also trained with text data including image retrieval, image-to-caption, and caption-to-image tasks.

### Model popularity

Given that the most used models were developed early in the computer vision field, we normalized the number of studies using a certain model to the square of the number of years since its first publication to approximate model popularity (Fig. 3b, and Supplementary Table 5, Supplementary Fig. 1d).

### Intelligent Explorer

The Intelligent Explorer allows users to search for papers based on specific study characteristics in ways that are not possible with conventional search methods. Moreover, a Visual Insights pane provides a quick overview of the problems investigated in different cancers. Parameters include cancer type, task type, machine learning task, data collection techniques, whether multiple cohorts were used, network type, dataset (if public), and class labels. After submission, the algorithm cleans the dataset to ensure integrity and relevance, incorporating one-hot encoding for categorical variables like data collection techniques and machine learning tasks. This preprocessing supports efficient data handling. The data is then customised to match user inputs via a form interface, ensuring contextually relevant analysis. The K-Nearest Neighbours (KNN) option considers best-performing studies and quality in addition to user-defined criteria while exact match aims to match the specified input by the user. A list of articles and visual summaries are retrieved and extended details are available for the user to download and analyze.

### Feature Ranking Tool

To help researchers with model design, we have also designed a Feature Ranking Tool, accessible from ‘Tools/Design assistant’. The tool ranks the importance of various implementation features based on various criteria such as cancer type or model type. Feature importance is computed using the ReliefF index and average performance. The features were evaluated independently to ensure the importance of correlated features does not affect the results.

### Dashboard development

HistoPathExplorer was implemented using the Flask Python web framework, and data was visualised using the Plotly package. HistoPathExplorer is updated monthly with new research articles. We also allow community submission through ‘Submit paper details page’. The latest update dates are shown on the website.

## Supplementary information

Supplementary figures and tables

Editorial Policy Checklist

## Data Availability

Data for all curated articles and the associated variables are available for downloading from https://histopathexpo.ai/.

## Code availability

The intelligent tools are developed using Python 3.10.6 and are publicly available on GitHub at https://github.com/sailem-group/histopathexpo.
