# Supplementary material for: AI in Histopathology Explorer for comprehensive analysis of the evolving AI landscape in histopathology
Source: NPJ Digit Med. 2025 Mar 12;8:156. doi: 10.1038/s41746-025-01524-2 (PMC11904230; doi:10.1038/s41746-025-01524-2)
Supplement: Supplementary file 1 — Supplementary figures and tables [file 41746_2025_1524_MOESM1_ESM.pdf]

Supplementary Figure 1: Overview plots from the dashboard. a) Associations between task types, network family, machine learning task, and cancer type. b) Hierarchical map showing the network categories, and families adopted in this work. CCL: Clustering-guided Contrastive Learning, DBN: Deep Belief Network, LSTM: Long-Short-Term Memory, RNN: Recurrent Neural Network, FCN: Fully Convolutional Network. c) Distribution of clinical tasks in IHC datasets. d) Timeline for neural network models identified in our study. Only the earliest version is shown if the models have multiple versions (e.g. ShuffleNet, and ShuffleNet_v2)

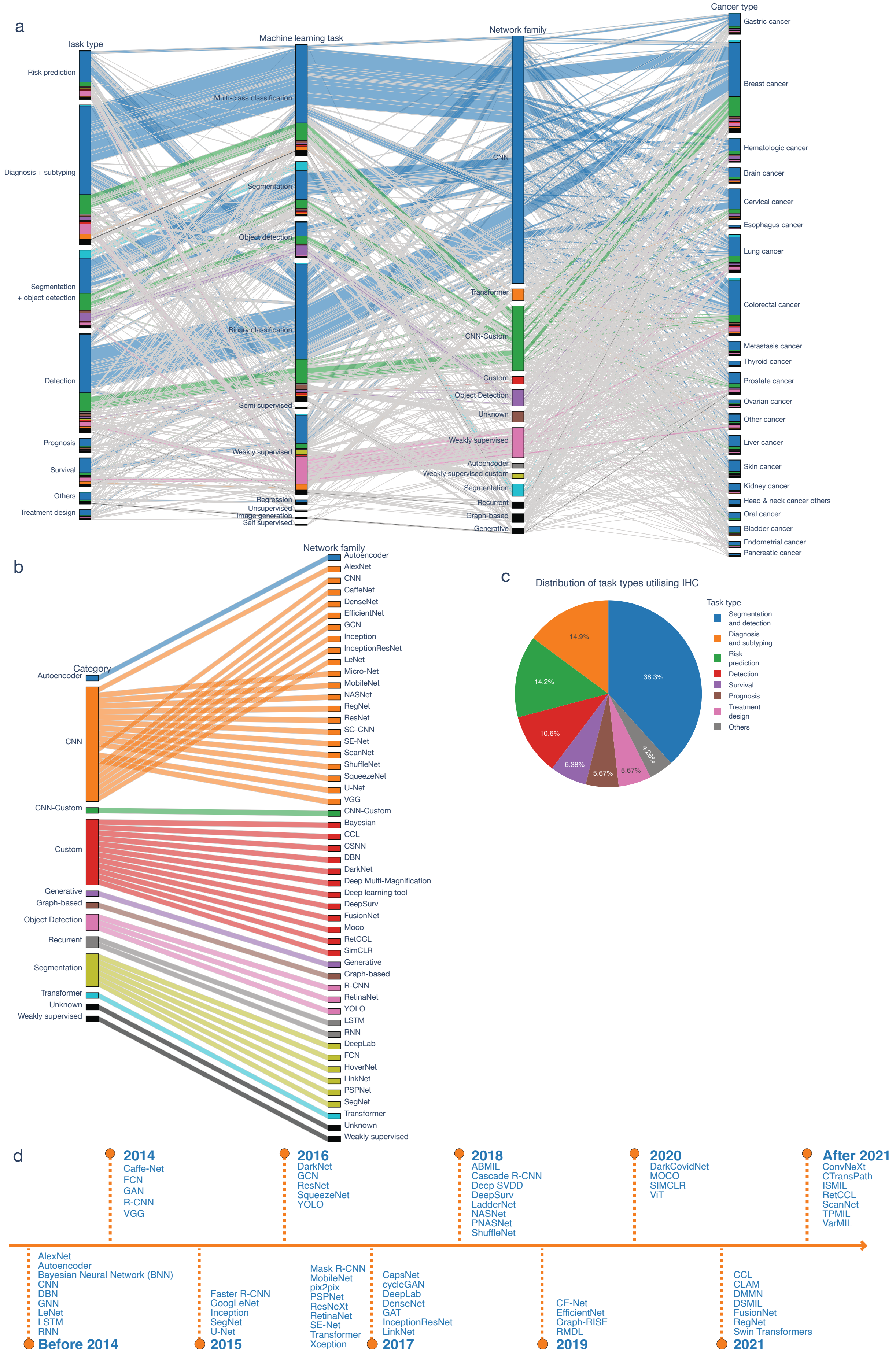

Supplementary Figure 2: Overview based on cancer types. a) Bar plot showing the number of papers by cancer type. b) Heat map showing the number of papers by clinical task, and cancer. Circle size, and colour indicate the number of papers. c) Overview of cancer type, and clinical task distribution.

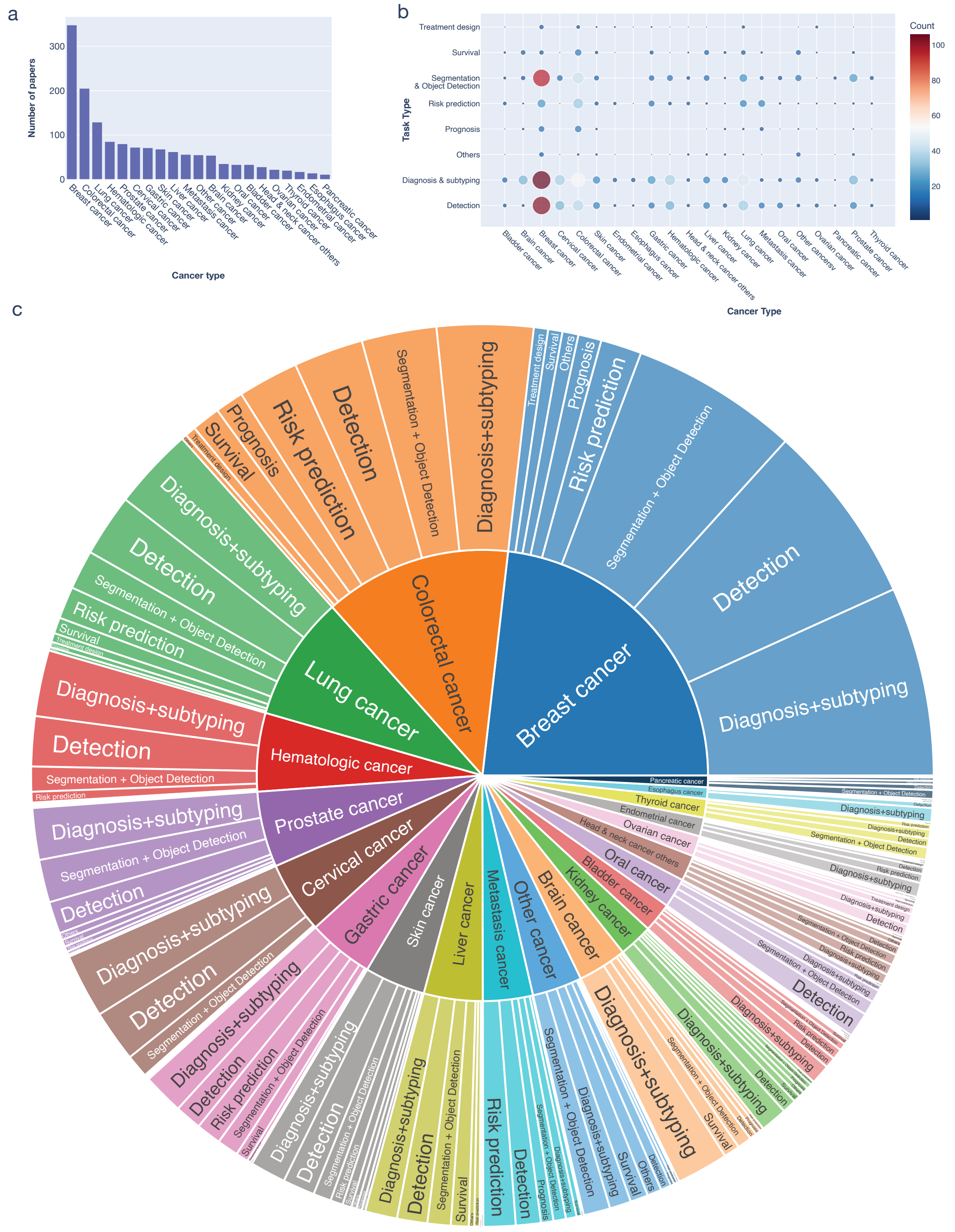

Supplementary Figure 3: Performance, and pre-processing methods. a-b) Median values of performance metrics for various neural network architectures when: all data types are considered (a), and when only H&E data are considered (b). The total number of papers included in the heat map for each family is depicted in the row above. c) Spearman correlation between data size, and performance metrics for the top used architectures. d) Spearman correlation between data size, and performance metrics when different model types are considered. e) Relationship between data size, and model accuracy. A bigger bubble indicates a larger data size. f) AUC when using different network depth ranges grouped by data collection technique. g-i) Performance of models utilising different augmentation techniques based on: AUC (g), sensitivity (h), and specificity (i). j) Frequency of usage of different data augmentation techniques. k) Average performance for models using different techniques to ensure class balance for each data collection technique.

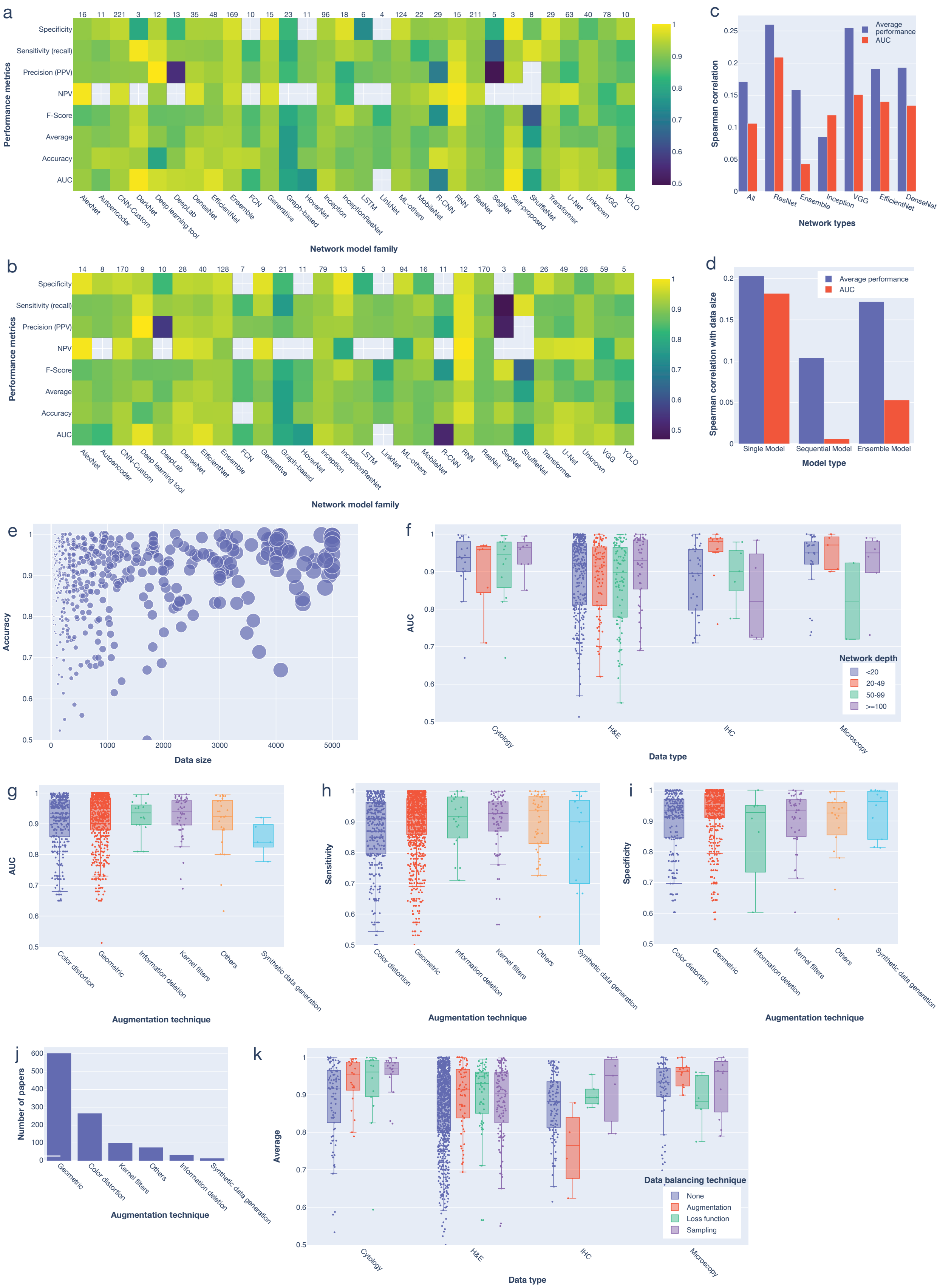

Supplementary Figure 4: Code, and Data Availability. a-b) The number of studies making their code, and data available by: clinical task (a) or by year (b). c) Proportion of papers reporting various metrics across different clinical tasks. d) A plot generated using the Network Tool showing the classification tasks investigated by different cancer types. The color of the cancer-type node indicate the average performance for that cancer across all studies.

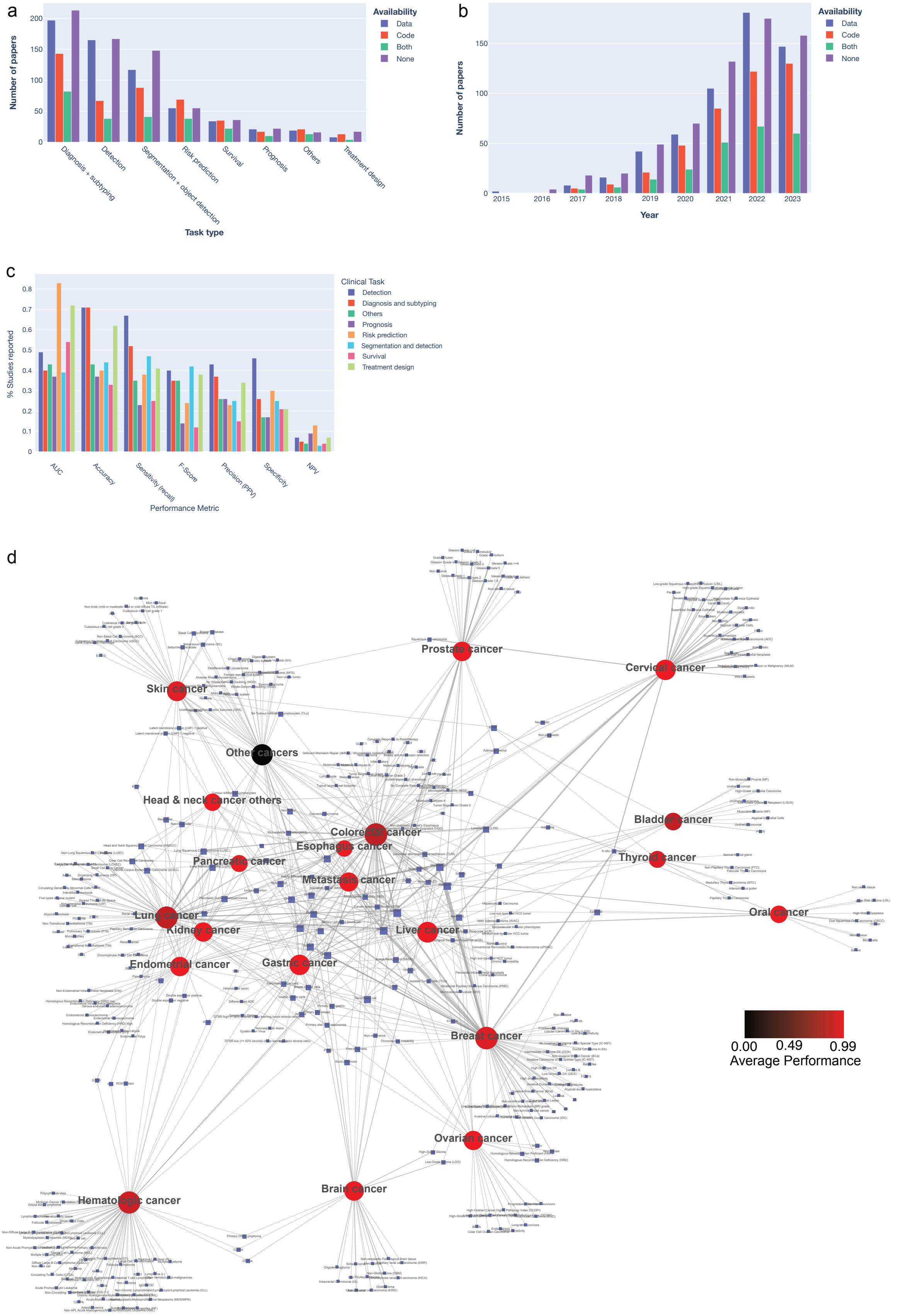

Supplementary Table 1. Data collection techniques

Category	Data Collection Techniques
Cytology	Liquid-Based cytology (LBC), pap smear, bone-marrow smear, cytology, hemacolor, tele-cytology, pleural fluids, cerebrospinal fluid, May-Grünwald Giemsa (MGG) stain, Diff-Quik, Argyrophilic Nucleolar Organizer Regions (AgNORs) stain, Wright, Wright-Giemsa, Giemsa
Cytometry	Cytometry, flow cytometry, holographic flow cytometry
Endomicroscopy	Endomicroscopy, high-resolution microendoscope, volumetric laser endomicroscopy
Genomic	DNA methylation, transcriptomics, genomics
H&E	Haematoxylin eosin saffron (HES) stain, haematoxylin and eosin (H&E) stain, hematoxylin phloxine saffron (HPS) stain.
Hyperpectral	Hyperspectral Imaging (HSI)
Immunohistochemistry	Immunohistochemical staining
Microscopy	Microscopy, Digital Holographic Microscopy (DHM), Mueller matrix microscope, multiphoton microscopy (MPM), CMOS sensor, Fourier Ptychographic (FP), micrographs, phonon microscopy, quantitative phase imaging, ultraviolet photoacoustic remote sensing microscopy, Second-Harmonic Generation (SHG) microscopy, Dual in situ hybridisation (DISH), in-situ hybridisation (ISH), Nonlinear Optical (NLO) microscopy
Multiplexed	Multiplexed
Other Histology Stain	Pricrosirius red, Massons trichrome, Masson Goldner
Other Histology Technique	Intraoperative Frozen Section (IFS)
Radiology	Computed Tomography (CT), Magnetic Resonance Imaging (MRI), ultrasound, X-rays
Spectroscopy	Infrared spectroscopic (IR) imaging, Raman, Stimulated Raman scattering (SRS) microscopy, spectroscopy, Mass spectrometry imaging (MSI), Stimulated Raman Histology (SRH), mid-infrared spectroscopic imaging (MIRSI), partial wave spectroscopic microscopy
Others	Clinical, text

Supplementary Table 2. Clinical task definitions

Task types	Definitions
Detection	Identifying signs of disease or detecting corresponding areas.
Prognosis	Predicting the likely outcome or course of a disease.
Risk prediction	Estimating the likelihood of a particular biological or pathological process, which could have impact on medical outcomes. E.g. gene mutation status, microsatellite instability status.
Segmentation or object detection	Segmenting or delineating a certain regions or cells in the tumour images or detecting the presence or locations of such objects within bounding boxes.
Diagnosis and subtyping	Predicting a cancer pre-condition, grade, or subtype.
Survival	Predicting overall survival or progression free survival.
Treatment design	Predicting treatment response or effectiveness

Supplementary Table 3. Public challenges for AI in histopathology

Year	Challenge	Origin	Data Type	Cancer	Tasks	Paper Count	References
2012	MITOS	France, Singapore	H&E	Breast	Mitosis detection	14	10.4103/2153-3539.112693
2013	AMIDA	Netherlands	H&E	Breast	Mitosis detection	5	10.1016/j.media.2014.11.010
2014	MITOS-ATYPIA-14	France, Singapore	H&E	Breast	Mitosis and Nuclear Atypia detection	15	http://ludo17.free.fr/mitos_atypia_2014/icpr2014_MitosAtypia_DataDescription.pdf
2014	Overlapping Cervical Cytology Image Segmentation challenge	Australia, China	Pap Smear	Cervical Cancer	Image Segmentation	1	10.1109/JBHI.2016.2519686
2015	Bioimaging 2015	Portugal	H&E	Breast	Tissue Classification	5	http://www.bioimaging2015.ineb.up.pt/ dataset.html.
2015	Gland Segmentation in Colon Histology Images (GLaS)	United Kingdom	H&E	Colon	Gland segmentation	18	10.1016/j.media.2016.08.008
2016	HER2 Challenge	United Kingdom	IHC	Breast	HER2 scoring	5	10.1111/his.13333
2016	TUPAC	Netherlands	H&E	Breast	Tumour proliferation scores	11	10.1016/j.media.2019.02.012
2016	CAMELYON16	Netherlands	H&E	Breast	Metastases detection in lymph node sections	20	10.1001/jama.2017.14585
2017	CAMELYON17	Netherlands	H&E	Breast	Metastases detection in lymph node sections	2	10.1109/TMI.2018.2867350
2017	PatchCamelyon	Netherlands	H&E	Breast	Metastases detection in lymph node sections	9	10.1001/jama.2017.14585 ; 10.48550/ARXIV.1806.03962
2018	BACH	Portugal	H&E	Breast	Classification of histology images	25	10.1016/j.media.2019.05.010
2018	MoNuSeg	USA, India	H&E	Multiple	Generalised nuclei segmentation	5	10.1109/TMI.2019.2947628
2019	DigestPath	China	H&E	Gastric mucosa and Intestine	Signet ring cell detection	3	10.1016/j.media.2022.102485

2019	LYSTO	Netherlands	IHC	Breast, liver, kidney, prostate, bladder, colon, stomach	Automatic assessment of lymphocytes	3	10.1109/JBHI.2023.3327489
2019	LYON	USA	IHC	Breast, Colon, and Prostate	Lymphocyte detection	3	10.1016/j.media.2019.101547
2019	C-NMC 2019	India	blood smear	Leukemia	identifying immature leukemic blasts	2	10.1016/j.medengphy.2022.103793
2019	ACDC Lung HP 2019	China	H&E	Lung	Lung cancer detection and classification	1	10.1109/JBHI.2020.3039741
2019	PAIP 2019	South Korea	H&E	Liver	Liver cancer segementation and viable tumor burden estimation	4	10.1016/j.media.2020.101854
2020	PAIP 2020	South Korea	H&E	Colorectal	Microsatellite Instability Prediction	2	10.1016/j.media.2023.102886
2020	Computational Precision Medicine Radiology-Pathology challenge on Brain Tumor Classification	USA	MRI, Histopathology	Brain	Automated classification of brain tumours	1	10.3389/fnins.2020.00027
2020	Prostate cANcer graDe Assessment (PANDA) Challenge	Netherlands, Sweden	H&E	Prostate	Prostate cancer grading	7	10.1038/s41591-021-01620-2
2020	ECDP 2020 HEROHE	Portugal	H&E	Breast	HER2 scoring	1	10.3390/jimaging8080213
2021	PAIP 2021	South Korea	H&E	Colon, Prostate and Pancreatobiliary tract	Detection of perineural invasion	1	PAIP2021 - Grand Challenge. *PAIP2021* Available at: https://paip2021.grand-challenge.org/.
2021	MIDOG 2021	Germany, Austria, Netherlands	H&E	Breast	Mitosis detection	2	10.1016/j.media.2022.102699
2022	MIDOG 2022	Germany, Austria, Netherlands	H&E	Breast	Mitosis detection	2	https://doi.org/10.48550/arXiv.2309.15589

Supplementary Table 4: Frequency of challenge datasets

Cancer	Count
Bladder	0
Brain	1
Breast	88
Cervical	1
Colorectal	25
CVA	0
Endometrial	0
Esophagus	0
Gastric	1
Head and Neck others	2
Kidney	0
Leukemia	2
Liver	5
Lung	5
Oral	0
Other	5
Ovarian	0
Pan-cancer	2
Pancreas	0
Prostate	14
Retina	0
Thyroid	1
Metastases	13
Dermalogical	2

Supplementary Table 5: Network publication year

Network name	Year	Full name
Autoencoder	1986	
BNN	1995	Bayesian Neural Network
RNN	1997	Recurrent Neural Network
LSTM	1997	Long-Short-Term Networks
CNN	1998	Convolutional Neural Network
LeNet	1998	
GNN	2005	Graph Neural Network
DBN	2006	Deep Belief Network
AlexNet	2012	
VGG	2014	
CaffeNet	2014	
GAN	2014	Generative Adversarial Network
R-CNN	2014	Region-Based Convolutional Neural Network
FCN	2014	Fully Convolutional Network
Inception	2015	
GoogLeNet	2015	
Faster R-CNN	2015	
U-Net	2015	
SegNet	2015	Segmentation Net
ResNet	2016	Residual Network
YOLO	2016	
DarkNet	2016	
SqueezeNet	2016	
GCN	2016	Graph Convolutional Network
Wide ResNet	2016	
ResNet50 v2	2016	
DeepLab	2017	
InceptionResNet	2017	

ViT	2020	Vision Transformer
Moco	2020	Momentum Contrast
DarkCovidNet	2020	
SimCLR	2020	SIMple framework for Contrastive Learning of visual
SC-CNN	2020	Spatially-Constrained Convolutional Neural Network
SwinTransformer	2021	Shifted Window Transformer
RegNet	2021	Self-Regulated Network
FusionNet	2021	
DMMN	2021	Deep Multi-Magnification Network
CCL	2021	Clustering-guided Contrastive Learning
DSMIL	2021	Dual-stream Multiple Instance Learning
CLAM	2021	Clustering-constrained Attention Multiple instance learning
ConvNeXt	2022	
ScanNet	2022	
VarMIL	2022	
CTransPath	2022	Transformer-based unsupervised Contrastive learning for
RetCCL	2023	Clustering-guided contrastive learning for whole-slide image
TPMIL	2023	Trainable Prototype Enhanced Multiple Instance Learning
ISMIL	2023	Intensive-sampling Multiple Instance Learning
Pretrained	2023	Pretrained foundation models trained on large scale data

Supplementary Table 6. Data augmentation techniques

Category	Definition	Types
Color distortion	Color Distortion involves a variety of techniques for altering the natural colors of an image. These changes can improve, adjust, or purposefully change how an image looks, making it suitable for various professional and creative uses.	Brightness, Gamma transformation, HSV variation, Constant, ZCA whitening, Inversion, Intensity variation, Photometric, Color jittering, Color shifting, Grayscale, Saturation, Contrast, Hue, Contrast limited adaptive histogram equalization (CLAHE), Maximum white balance change, Solarization, RGB augumentation, Normalisation, HED color space perturbation, Chroma, FancyPCA
Geometric	Geometric category involves a wide range of techniques focused on adjusting an image's layout. This includes rotating, flipping, cropping, and changing perspective. These tools allow for the resizing, shaping, and altering of images to change how they are viewed, create optical illusions, or tailor dimensions for particular analytical or design purposes.	Rotation, Flipping, Cropping, Optical or grid distortion, Warping, Padding, Perspective transformation, Skewing, Elastic transformation, Distortion, Rigid deformation, Transposed, Embossing, Deformation, Scaling, Zooming, Shifting, Shearing, Affine transformation, Translation, Resizing, Barrel and pincushion distortion
Information deletion	Information Deletion focuses on methods designed to intentionally eliminate or obscure parts of image data. Through techniques like dropout, cutout, and the use of Gaussian noise or JPEG compression, these processes remove, substitute, or degrade elements within images. This simulates different kinds of data loss or distortion, aiming to improve model resilience or expand data for training purposes.	Cutmix, Progressive sprinkles, Cutout, JPEG compression, Salt and pepper noise

Kernel filters	Kernel Filters involves techniques using convolutional kernels to change an image's appearance, especially its texture and sharpness. Methods like Gaussian blur, sharpening, and median filtering are applied to either soften, highlight edges, or add specific blur and noise effects. These approaches are valuable for multiple objectives, including minimizing noise and details for initial processing, accentuating features, or mimicking camera actions and focus adjustments.	Gaussian blurring, Gaussian noise, Sharpening, Edge sharpening, Blurring, Gaussian filtering, Noise, Motion blurring, Median blurring, Glass blurring
Others	The 'Others' category captures a broad mix of data augmentation techniques and methods that fall outside standard classifications. It includes experimental approaches, general random augmentations, real-time techniques, and tools for various transformations, aiming to increase dataset diversity or explore new augmentation strategies.	Unknown, Self-proposed, Random augmentation, On-the-fly data augmentation, Shuffle, ImageDataGenerator
Synthetic data generation	Synthetic Data Generation involves techniques such as Generative Adversarial Networks (GANs), CycleGANs, and autoencoders to produce artificial data closely resembling real datasets. Utilizing deep learning, these methods generate unique data examples nearly identical to genuine ones, supporting applications in data augmentation, privacy protection, and the training of machine learning models when real data is limited or confidential.	GANs, CCG-taming transformers, Autoencoder, Mixup, Mosaic, CycleGANs

Supplementary Table 7: Data balancing techniques

Category	Definition	Methods
Augmentation	Augmentation involves strategies like data augmentation, downsampling, upsampling, and distributed label augmentation to achieve a balanced dataset. These techniques enhance data variety and adjust distribution to support more robust and accurate machine learning models.	Data augmentation, Distributed label augmentation
Loss function	Loss Function is designed to refine model accuracy by addressing data imbalances and specific evaluation needs, these functions play a crucial role in optimizing machine learning outcomes.	Weighted cross entropy, Harmonic mean, Focal loss, Online hard example mining (OHEM)
Sampling	Sampling category, including Equal, Balanced, and Weighted Sampling, as well as techniques like SMOTE and Undersampling, focus on achieving a balanced data distribution. These methods vary from generating synthetic samples to adjusting class representation, ensuring diverse and equitable data for model training and improved performance.	Equal sampling, Balanced sampling, Synthetic minority over-sampling technique (SMOTE), Undersampling, Weighted sampling, Differentiate enhancement-random sampling (DEES), Stratified sampling, Tomek Links undersampling, Oversampling, Downsampling, Upsampling, Dynamic sampling

Supplementary Table 8: Explainability techniques

Category	Definition	Methods
Heatmap	Utilized color-coded maps to highlight the areas that significantly influence the decision-making process of a model.	Class Activation Mapping (CAM), Grad-CAM, Grad-CAM++, Guided Grad-CAM, ScoreCAM, SmoothGrad, Occlusion map, Integrated Gradients, Probability heatmap, Uncertainty heatmap, Full-Grad, Guided backpropagation (GB), saliency map, Global Normalised-CAM, Eigen-CAM
Dimensionality reduction	Converting feature vectors extracted by the models to 2D data points to gain a better understanding of the predictions.	Principal Component Analysis (PCA), Uniform Manifold Approximation and Projection (UMAP), t-Distributed Stochastic Neighbor Embedding (t-SNE), bh-SNE
Attention-based	Enabling models to weigh different parts of the input image, focusing on more relevant features while suppressing less useful ones.	attention map, attention mechanism, layer-wise relevance propagation (LRP) rollout
Task-specfic knowledge	Generating interpretable features that could be generated by a deep learning model, which could be feed into another machine learning or deep learning model as input.	task-specific knowledge
Feature-based	Attributing significance to specific features to better understand the model's predictions.	Feature based, Feature importance, Feature Activation Map (FAM), LIME, SHAP, Deep-dream
Concept-based	Presenting 'concepts' based on the extracted features that are more human-understandable than pixels.	Concept based, Automatic Concept-based Explainations (ACE), Concept Activation Vectors (CAV)
Feature integration	Incorporating other interpretable features can be extracted from the images, such as morphological and cell type data, into the model training.	Feature integration

Supplementary Table 9: HER2 articles

ID	Title	Publication date	Quality index	Cancer type	Clinical task	Network type	Data collection technique	Class label	Data size	Performance average	Performance AUC	Performance sensitivity	Performance specificity	Performance metrics (3 or more)	Data availability	Data source	Code availability	Code source	Methodology	External validation	Benchmarking	Implementation details
253	Deep convolutional neural networks for automatic classification of gastric carcinoma using whole slide images in digital histopathology.	2017	3	Gastric cancer	Diagnosis and subtyping	Self-proposed	H&E	HER2, Non-tumor	231000 (Patches)	0.699	-	-	-	FALSE	FALSE	-	FALSE	-	TRUE	FALSE	TRUE	TRUE
747	Breast Cancer Molecular Subtype Prediction on Pathological Images with Discriminative Patch Selection and Multi-Instance Learning.	2022	3	Breast cancer	Diagnosis and subtyping	ResNet50	H&E	Basal-like, HER2, Luminal A, Luminal B	1254 (Whole slide images)	0.675	-	0.722	-	TRUE	FALSE	-	FALSE	-	TRUE	FALSE	TRUE	FALSE
792	Deep learning identifies morphological features in breast cancer predictive of cancer ERBB2 status and trastuzumab treatment efficacy.	2021	2	Breast cancer	Detection	SE-ResNetxt50+32x4d	H&E	HER2	1047 (Tissue microarray)	-	-	-	-	FALSE	FALSE	-	FALSE	-	TRUE	TRUE	FALSE	FALSE
799	Deep Learning to Estimate Human Epidermal Growth Factor Receptor 2 Status from Hematoxylin and Eosin-Stained Breast Tissue Images.	2020	3	Breast cancer	Diagnosis and subtyping	U-Net, Self-proposed	H&E	HER2	97 (Whole slide images)	0.806	0.76	0.87	0.6	TRUE	FALSE	https://portal.gdc.cancer.gov	FALSE	-	TRUE	TRUE	FALSE	FALSE
800	Deep learning trained on hematoxylin and eosin tumor region of Interest predicts HER2 status and trastuzumab treatment response in HER2+ breast cancer.	2021	3	Breast cancer	Diagnosis and subtyping	InceptionV3	H&E	HER2	188 (Whole slide images)	0.82	0.82	-	-	FALSE	FALSE	https://portal.gdc.cancer.gov	FALSE	upon req	TRUE	TRUE	FALSE	TRUE
1052	HAHNet: a convolutional neural network for HER2 status classification of breast cancer.	2023	4	Breast cancer	Diagnosis and subtyping	InceptionV3	H&E	HER2	4873 (Patches)	0.945	0.99	0.925	-	TRUE	FALSE	https://bupt-ai-cz.github.io/BCI/	FALSE	-	TRUE	FALSE	TRUE	TRUE
1068	Data-Efficient Computational Pathology Platform for Faster and Cheaper Breast Cancer Subtype Identifications: Development of a Deep Learning Model.	2023	4	Breast cancer	Diagnosis and subtyping	MIL, ResNet50, SimCLR	H&E	AR, ER, HER2, Ki67, PR	187921 (Patches)	0.858	0.858	-	-	FALSE	FALSE	-	TRUE	https://github.com/nus-mornin-lab/3DHistoNet	TRUE	FALSE	TRUE	TRUE

Supplementary Table 10. Papers aimed at microsatellite instability detection using H&E data

Study	Year	Quality index	Cancer Type	Explainability	Network type	Data size	Performance average	AUC	Sensitivity	Specificity	Age	Dataset	Dataset country	Data availability	Code availability	Performance metrics (3 or more)	Methodology	External validation	Benchmarking	Implementation detail
Niehues et al.	2023	5	Colorectal	Grad-CAM	Self-proposed	188 (Whole slide images)	0.940	0.940	-	-	-	DACHS, QUASAR	United Kingdom, Germany	✗	✓	✗	✓	✓	✓	✓
Bustos et al.	2021	3	Colorectal	SHAP, PCA	Swin Transformer	13689 (Patient)	0.845	0.900	0.870	0.883	(27, 101)	private-set	Spain	✗	✗	✓	✓	✗	✗	✗
Krause et al.	2021	4	Colorectal	t-SNE, Occlusion Map	HoverNet, U-Net, Self-proposed	86 (Patient)	0.777	0.777	-	-	-	TCGA, NLCS	USA	✓	✓	✗	✗	✗	✗	✓
Su et al.	2022	5	Colorectal	Grad-CAM, probability heatmap	ResNet34	100000 (Patches)	0.861	-	0.833	0.885	-	Su et al	China	✓	✓	✓	✓	✗	✗	✓
Echle et al.	2020	5	Colorectal	-	Self-proposed	7177 (Whole slide images)	0.847	0.920	0.950	0.670	-	TCGA, MSI-Detect, DACHS, QUASAR, NLCS	USA, Germany, United Kingdom, Netherlands	✓	✓	✓	✗	✓	✗	✓
Lee et al.	2021	5	Colorectal	-	Swin Transformer	1527 (Whole slide images)	0.861	0.861	-	-	(26, 90)	TCGA, SMH	USA, South Korea	✓	✓	✗	✓	✓	✗	✓
Kather et al.	2019	5	Colorectal	-	MIL, DenseNet121	441 (Whole slide images)	0.840	0.840	-	-	-	TCGA, DACHS	Germany, USA	✓	✓	✗	✓	✓	✗	✓
Qiu et al.	2022	4	Colorectal	-	ResNet152	1409 (Whole slide images)	0.952	0.952	-	-	-	NCT-CRC-HE-100K	Germany	✓	✗	✗	✓	✗	✓	✗
Jiang et al.	2022	4	Colorectal	-	MIL, ResNet18	429 (Whole slide images)	0.895	0.889	0.909	0.951	-	TCGA, in-house, PAIP2020	China, USA, South Korea	✗	✓	✓	✗	✓	✗	✓
Schirris et al.	2022	6	Colorectal	-	ABMIL, ResNet50, CCL	2190 (Whole slide images)	0.740	0.870	-	-	-	TCGA	USA	✓	✓	✗	✓	✗	✓	✓
Lou et al.	2022	3	Colorectal	-	U-Net, VGG11	547 (Whole slide images)	0.848	0.885	-	-	-	private-set	China	✗	✗	✗	✗	✗	✓	✓
Fujii et al.	2022	5	Colorectal	knowledge	Self-proposed, InceptionV3	584 (Whole slide images)	0.893	0.893	-	-	-	SCRUM-Japan GI-SCREEN	Japan	✓	✓	✗	✓	✗	✗	✓
Saldanha et al.	2022	4	Colorectal	probability heatmap	HoverNet	428 (Whole slide images)	0.809	0.809	-	-	-	private-set, TCGA	United Kingdom, USA	✗	✓	✗	✗	✗	✓	✓
Gerwert et al.	2023	3	Colorectal	-	MobileNet, InceptionV3	46311 (Patches)	0.876	0.920	0.870	0.870	-	private-set	Germany	✗	✗	✓	✓	✗	✗	✗
Frank	2023	4	Colorectal	-	ResNet34	188 (Whole slide images)	0.810	-	-	-	-	PAIP2020	South Korea	✓	✓	✗	✗	✗	✓	✓
Muti et al.	2021	3	Colorectal	-	ResNet18	989938 (Patches)	0.843	0.843	-	-	(55, 78.3)	TCGA	USA	✗	✓	✗	✗	✗	✗	✓
Schrammen et al.	2021	6	Colorectal	probability heatmap	MIL, ShuffleNet	2039 (Whole slide images)	0.838	0.909	0.975	0.878	-	DACHS, private-set	Germany, United Kingdom	✗	✓	✓	✓	✓	✓	✓
Cao et al.	2020	6	Colorectal	feature integration, probability heatmap	ResNet18	360 (Patient)	0.885	0.885	-	-	-	TCGA	USA	✓	✓	✗	✓	✓	✓	✓
Yamashita et al.	2021	4	Colorectal	probability heatmap	MobileNetV2	66578 (Patches)	0.786	0.779	0.760	0.666	-	private-set, TCGA	USA	✗	✗	✓	✓	✓	✓	✗
Zhu et al.	2022	3	Colorectal	Grad-CAM	CycleGAN, ShuffleNet	150000 (Patches)	0.817	0.817	-	-	-	TCGA	USA	✓	✗	✗	✗	✓	✗	✓
Wagner et al.	2023	5	Colorectal	attention map	VarMIL, ResNet18, Self-proposed, SimCLR	192314 (Patches)	0.990	-	0.990	-	-	TCGA, CPTAC, Northern Ireland Biobank, MCO dataset	USA, Northern Ireland, Australia	✗	✓	✗	✓	✗	✓	✓
Awan et al.	2022	2	Colorectal	-	HoverNet, InceptionV3	534771 (Patches)	0.960	0.960	-	-	-	private-set	United Kingdom	✗	✗	✗	✓	✓	✗	✗
Saillard et al.	2023	4	Colorectal	-	Chowder	1091 (Whole slide images)	0.767	0.880	0.970	0.450	-	PAIP2020, TCGA	South Korea, USA	✓	✓	✓	✗	✓	✗	✓
Ke et al.	2021	1	Colorectal	-	Self-proposed	747 (Whole slide images)	0.939	0.939	-	-	-	TCGA, CRC	USA	✓	✗	✗	✗	✗	✗	✗
Saillard et al.	2021	5	Colorectal	-	ResNet50, DeepMIL, Chowder	555 (Whole slide images)	0.920	0.920	-	-	-	TCGA	USA	✓	✗	✗	✓	✓	✓	✓
Echle et al.	2022	7	Colorectal	-	ResNet18	8343 (Patches)	0.848	0.960	0.950	0.890	-	TCGA, DACHS, QUASAR, NLCS, MECC	Germany, USA, Netherlands, Israel, United Kingdom	✓	✓	✓	✓	✓	✓	✓
Wang et al.	2022	6	Gastric	UMAP	Self-proposed, InceptionV3	177 (Whole slide images)	0.811	0.811	-	-	-	TCGA	USA	✓	✓	✗	✓	✗	✓	✓
Rubinstein et al.	2022	3	Gastric	task-specific knowledge	ResNet18	467 (Whole slide images)	0.700	0.700	-	-	-	TCGA	USA	✓	✗	✗	✓	✗	✗	✗
Guo et al.	2023	5	Gastric	Grad-CAM	ShuffleNet	443 (Whole slide images)	0.910	0.910	-	-	-	TCGA	USA	✓	✓	✗	✗	✗	✓	✓
Lee et al.	2022	4	Gastric	t-SNE	Self-proposed	2101 (Whole slide images)	0.867	0.902	0.917	0.833	-	TCGA, SSMH-dataset	USA, South Korea	✗	✓	✓	✗	✓	✗	✓
He et al.	2024	2	Gastric	Grad-CAM	EfficientNetB1	334938 (Patches)	0.790	0.790	-	-	-	TCGA	USA	✓	✗	✗	✓	✗	✓	✗
Bilal et al.	2021	4	Colorectal	feature based, probability heatmap	ResNet18	1033 (Whole slide images)	0.860	0.860	-	-	-	TCGA	USA	✓	✗	✗	✓	✓	✗	✓
