# Supplementary material for: AI in Histopathology Explorer for comprehensive analysis of the evolving AI landscape in histopathology
Source: NPJ Digit Med. 2025 Mar 12;8:156. doi: 10.1038/s41746-025-01524-2 (PMC11904230; doi:10.1038/s41746-025-01524-2)
Supplement: Supplementary file 2 — Editorial Policy Checklist [file 41746_2025_1524_MOESM2_ESM.pdf]

nature portfolio

Corresponding author(s): Heba Sailem

Last updated by author(s): 6/02/2025

Editorial Policy Checklist

This form is used to ensure compliance with Nature Portfolio editorial policies related to research ethics and reproducibility. For further information, please see our editorial policies site. All relevant questions on the form must be answered.

Competing interests

Policy information about competing interests

In the interest of transparency and to help readers form their own judgements of potential bias, Nature Portfolio journals require authors to declare any competing financial and/or non-financial interest in relation to the work described in the submitted manuscript.

Competing interests declaration

- [x] We declare that none of the authors have competing financial or non-financial interests as defined by Nature Portfolio.
- [ ] We declare that one or more of the authors have a competing interest as defined by Nature Portfolio.

Authorship

Policy information about authorship

Prior to submission all listed authors must agree to all manuscript contents, the author list and its order and the author contribution statements. Any changes to the author list after submission must be approved by all authors.

- [x] We have read the Nature Portfolio Authorship Policy and confirm that this manuscript complies.

Large Language Models (LLMs), such as ChatGPT, do not currently satisfy our authorship criteria. Notably an attribution of authorship carries with it accountability for the work, which cannot be effectively applied to LLMs. Use of an LLM should be properly documented in the Methods section (and if a Methods section is not available, in a suitable alternative part) of the manuscript.

- [ ] We confirm that the author list of this manuscript does not include any Large Language Models (LLMs).

Policy information about Authorship: inclusion & ethics in global research

All authors are encouraged to provide an "Inclusion & Ethics" statement where relevant.

- [ ] We have provided an "Inclusion & Ethics" statement.

Data availability

Policy information about availability of data

Data availability statement

All manuscripts must include a data availability statement. This statement should provide the following information, where applicable:

- Accession codes, unique identifiers, or web links for publicly available datasets
- A description of any restrictions on data availability
- For clinical datasets or third party data, please ensure that the statement adheres to our policy

- [x] We have provided a full data availability statement in the manuscript.

Mandated accession codes (where applicable)

Confirm that all relevant data are deposited into a public repository and that accession codes are provided.

- [ ] All relevant accession codes are provided
- [ ] Accession codes will be available before publication
- [x] No data with mandated deposition

Code availability

Policy information about [availability of computer code]

Code availability statement

For all studies using custom code or mathematical algorithm that is deemed central to the conclusions, the manuscript must include a statement under the heading "Code availability" describing how readers can access the code, including any access restrictions. Code availability statements should be provided as a separate section after the data availability statement but before the References.

[x] We have provided a full code availability statement in the manuscript

Data presentation

For all data presented in a plot, chart or other visual representation confirm that:

n/a	Confirmed	
[ ]	[x]	Individual data points are shown when possible, and always for $n \leq 10$
[ ]	[x]	The format shows data distribution clearly (e.g. dot plots, box-and-whisker plots)
[ ]	[x]	Box-plot elements are defined (e.g. center line, median; box limits, upper and lower quartiles; whiskers, 1.5x interquartile range; points, outliers)
[ ]	[x]	Clearly defined error bars are present and what they represent (SD, SE, CI) is noted

Image integrity

Policy information about [image integrity]

[x] We have read Nature Portfolio's image integrity policy and all images comply.

Unprocessed data must be provided upon request. Please double-check figure assembly to ensure that all panels are accurate (e.g. all labels are correct, no inadvertent duplications have occurred during preparation, etc.).

Where blots and gels are presented, please take particular care to ensure that lanes have not been spliced together, that loading controls are run on the same blot, and that unprocessed scans match the corresponding figures.

Additional policy considerations

Some types of research require additional policy disclosures. Please indicate whether each of these apply to your study. If you are not certain, please read the appropriate section before selecting a response.

Does not apply	Involved in the study	
[x]	[ ]	Macromolecular structural data
[x]	[ ]	Unique biological materials
[x]	[ ]	Research animals and/or animal-derived materials that require ethical approval
[x]	[ ]	Human embryos, gametes and/or stem cells
[x]	[ ]	Human research participants
[x]	[ ]	Clinical data
[x]	[ ]	Archaeological, geological, and palaeontological materials

Macromolecular structural data

Po x cy information about [special considerations] for specific types of data

Validation report

[ ] We have provided an official validation report from [wwPDB] for all macromolecular structures studied.

Biological materials

Policy information about [availability of materials]

Obtaining biological materials

[ ] We have described these restrictions in the manuscript. [ ] We have described how to obtain all materials in the manuscript.

Research animals

Policy information about studies involving animals; ARRIVE guidelines recommended for reporting animal research

Ethical compliance

☐ We have complied with all relevant ethical regulations and include a statement affirming this in the manuscript.

Ethics committee

☐ We have disclosed the name(s) of the board and institution that approved the study protocol in the manuscript.

Human embryos, gametes and stem cells

Policy information about studies involving human embryos, gametes and stem cells

Manuscripts involving the use of human embryos, gametes or stem cells must include an ethics statement that provides the following information:

- The institutional and/or licensing committee(s) that approved the study protocol
- Confirmation that informed consent was obtained from all recipients and/or donors of cells or tissues
- The conditions for donating materials for the research

☐ We have read the Nature Portfolio policy on human embryos, gametes and stem cells and have complied with policy requirements.

Human research participants

Policy information about studies involving human research participants

Ethical compliance

☐ We have complied with all relevant ethical regulations and include a statement affirming this in the manuscript.

Ethics committee

Confirm that the manuscript states the name(s) of the board and/or institution that:

☐ Approved the study protocol -OR- ☐ Provided guidelines for study procedures (if protocol approval is not required)

Informed consent

☐ We have obtained informed consent from all participants and this is noted in the manuscript.

Identifiable images

For publication of identifiable images of research participants, confirm that consent to publish was obtained and is noted in the Methods.

Authors must ensure that consent meets the conditions set out in the Nature Portfolio participant release form.

☐ Yes ☐ No identifiable images of human research participants

Clinical studies

Policy information about clinical studies

Clinical trial registration

☐ We have provided the trial registration number from ClinicalTrials.gov or an equivalent agency in the manuscript.

Phase 2 and 3 randomized controlled trials

We have provided the CONSORT checklist with your submission.

☐ Yes ☐ No ☐ Not a phase 2/3 randomized controlled trial

Tumor marker prognostic studies

We have followed the REMARK reporting guidelines.

☐ Yes ☐ No ☐ Not a tumor marker prognostic study

Archaeological, geological, and palaeontological materials

Policy information about studies involving archaeological, geological, and palaeontological materials

☐ We affirm that archaeological, geological, and palaeontological materials samples were collected (and, where applicable, exported) in a responsible manner and in accordance with relevant permits and local laws, and that this information is detailed within the manuscript.

I certify that all the above information is complete and correct.

Typed signature ______________________ Date 29/01/2024

This checklist template is licensed under a Creative Commons Attribution 4.0 International License, which permits use, sharing, adaptation, distribution and reproduction in any medium or format, as long as you give appropriate credit to the original author(s) and the source, provide a link to the Creative Commons license, and indicate if changes were made. The images or other third party material in this article are included in the article's Creative Commons license, unless indicated otherwise in a credit line to the material. If material is not included in the article's Creative Commons license and your intended use is not permitted by statutory regulation or exceeds the permitted use, you will need to obtain permission directly from the copyright holder. To view a copy of this license, visit http://creativecommons.org/licenses/by/4.0/
